# Characterization of oncohistone H2B variants in *Schizosaccharomyces pombe* reveals a key role of H2B monoubiquitination deficiency in genomic instability by altering gene expression

**DOI:** 10.1093/femsyr/foaf027

**Published:** 2025-05-22

**Authors:** Guangchun Lu, Li Liu, Mitchell Opoku, Ruifan Zhu, Haiyang Wang, Gang Feng

**Affiliations:** Jiangsu Key Laboratory for Pathogens and Ecosystems, College of Life Sciences, Nanjing Normal University, Nanjing 210023, China; Jiangsu Key Laboratory for Pathogens and Ecosystems, College of Life Sciences, Nanjing Normal University, Nanjing 210023, China; Jiangsu Key Laboratory for Pathogens and Ecosystems, College of Life Sciences, Nanjing Normal University, Nanjing 210023, China; Jiangsu Key Laboratory for Pathogens and Ecosystems, College of Life Sciences, Nanjing Normal University, Nanjing 210023, China; Jiangsu Key Laboratory for Pathogens and Ecosystems, College of Life Sciences, Nanjing Normal University, Nanjing 210023, China; Jiangsu Key Laboratory for Pathogens and Ecosystems, College of Life Sciences, Nanjing Normal University, Nanjing 210023, China

**Keywords:** *Schizosaccharomyces pombe*, fission yeast, oncohistone H2B, H2B monoubiquitination, gene expression, genomic instability

## Abstract

Various amino acid substitutions commonly occur at one residue of a histone in human cancers, but it remains unclear whether these histone variants have distinct oncogenic effects and mechanisms. Our previous modeling study in the fission yeast *Schizosaccharomyces pombe* demonstrated that the oncohistone mutants H2BG52D, H2BD67N, and H2BP102L cause the homologous recombination defects and genomic instability by compromising H2B monoubiquitination (H2B^ub^). However, it is unknown whether other amino acid changes at the H2B-Gly52/Asp67/Pro102 residues influence H2B^ub^ levels and whether they cause genomic instability by altering H2B^ub^-regulated gene expression. Here, we construct diverse oncomutants at the sole H2B gene *htb1-Gly52/Asp67/Pro102* sites in *S. pombe* and study their impacts on genotoxic response, H2B^ub^ levels, and gene expression. Interestingly, the oncomutants *htb1-G52D, htb1-D67N*, and *htb1-P102L* exclusively exhibit significant genotoxic sensitivity, reduced H2B^ub^ levels, and altered gene expression. These defects can be rescued by restoring H2B^ub^ levels with the deletion of the H2B deubiquitinase *ubp8^+^*. These strong genetic correlations suggest that H2B^ub^ deficiency plays a determinant role in the genomic instability of *htb1-Gly52/Asp67/Pro102* oncomutants and that the alteration of gene expression due to reduced H2B^ub^ levels is a novel mechanism underlying the genomic instability caused by *htb1-G52D, htb1-D67N*, and *htb1-P102L* oncomutations.

## Teaser

Characterization of various histone H2B oncomutants using fission yeast as a model demonstrates that H2B monoubiquitination defect plays a crucial role in genomic instability by altering gene expression.

## Introduction

The missense mutations in a single copy of histone genes are frequently found in human cancers, and the encoded proteins are described as oncohistones (Mohammad and Helin 2017, Qiu et al. 2018). A residue of an oncohistone can be substituted for various amino acids with different frequencies (Funato and Tabar 2018). In the case of canonical oncohistones, H3K27M (lysine-to-methionine), H3G34R/V/W/L (glycine-to-arginine/valine/tryptophan/leucine), and H3K36M mutants occur most frequently in pediatric cancers (Liu et al. 2014, Kallappagoudar et al. 2015, Wan et al. 2018). Their common oncogenic mechanism is the perturbation of posttranslational modifications (PTMs) of histone H3 (Weinberg et al. 2017, Lowe et al. 2019, Espinoza Pereira et al. 2023). Two main consequences of perturbed histone PTMs are alteration in gene expression and genomic instability, both of which can promote oncogenesis (Dabin et al. 2024, Selvam et al. 2024, Yadav et al. 2024).

The H3K27M mutant, which occurs in 78% of diffuse intrinsic pontine gliomas, and the H3K27I (lysine-to-isoleucine) reduce the global H3K27me3 levels *in trans*, whereas the other 18 amino acid substitutions of H3K27 have no such effect (Lewis et al. 2013). However, the reduction in H3K27me3 levels and its impact on gene expression are not uniform across the genome (Chan et al. 2013). H3K27me3 abundance is reduced in weak PRC2 complex target genes and enhances their expression; however, H3K27me3 is retained in strong PRC2 target genes and silences genes (Sahu and Lu 2022). Therefore, the influence of H3K27M/I on gene expression in cancer may not be simply attributed to the loss of H3K27 methylation or PRC2 activity. In addition, H3K27M reduces homologous recombination (HR) and increases replication stress (Caeiro et al. 2024). The H3K36M mutant, which is identified in 95% of chondroblastomas, and the H3K36I both lead to reduced levels of global H3K36me2/3 and elevated levels of H3K27me2/3 *in trans*. However, other oncohistone H3K36R and nononcohistone H3K36L/A (lysine-to-leucine/alanine) have no such effects (Lu et al. 2016). The genome-wide altered gene expression does not appear to be determined mainly by the loss of H3K36me2/3. Instead, the gain and redistribution of H3K27me3 may play a more significant role in the gene expression profile of the H3K36M mutant (Sahu and Lu 2022). Moreover, H3K36M decreases the activity of HR repair for DNA breaks (Caeiro et al. 2024), and H3K36R is also defective in the response to DNA breaks (Zhang et al. 2023). Among the H3G34 oncomutants, the H3G34R/V are found in 20% of pediatric high-grade glioma, whereas H3G34W/L occur in 92% of giant cell tumors of the bone. These mutants decrease the abundance of H3K36me2/3 *in cis* (Chan et al. 2013, Lewis et al. 2013, Fang et al. 2018, Shi et al. 2018). However, the aberrant gene expression in H3G34W can be restored by H3K27R (lysine-to-arginine), implying that the gain of H3K27me3 levels, rather than the loss of H3K36me3, could be its direct mechanism (Jain et al. 2020). Moreover, H3G34R attenuates HR and mismatch repair, and H3G34W compromises DNA repair via nonhomologous end joining (Caeiro et al. 2024). Collectively, the influences of canonical H3 oncohistones on gene expression and genome stability vary with different amino acid substitutions, which could not be attributed solely to changes in H3 PTMs.

In addition, many noncanonical oncomutants occur at both the tails and the globular domains of histones (Bennett et al. 2019). In contrast to the high frequency and tissue specificity of canonical H3 oncohistones in pediatric cancers, noncanonical histone oncomutants occur widely in ~4% of adult common tumors, and few are significantly associated with a particular type of tumor (Nacev et al. 2019). These noncanonical oncohistones affect chromatin states and remodeling (Mitchener and Muir 2022). However, the functional impacts and mechanisms of different amino acid substitutions at residues of noncanonical oncohistones remain largely unclear. A previous study showed that H3K9M rather than H3K9R (lysine-to-arginine) diminishes the overall amount of H3K9me2/3 (Lewis et al. 2013). In addition, both acetylation-deficient H4K91R and acetylation-mimic H4K91Q (lysine-to-glutamine) abolish the acetylation and ubiquitination of H4K91, resulting in genomic instability and abnormal development H4K91M mutation (Tessadori et al. 2017). Taken together, different amino acid substitutions in various noncanonical oncohistones could also exhibit distinct effects and mechanisms.

Therefore, comprehensive variant studies of a particular histone residue are required to gain mechanistic insights into the oncogenic effects of an oncohistone and determine whether it acts as a driver or passenger in cancer development. This poses a challenge to the aforementioned research conducted in mammalian cells, but can be readily achieved by modeling in yeast, which shares ~90% sequence identity with human H3/H4 and 70% identity with human H2A/H2B. In contrast to many copies of histone genes in humans, both budding and fission yeast contain only a few copies, which could amplify the oncomutant effects, facilitate genetic analysis by performing rapid and unbiased screens, and clarify the functional consequences of oncohistones (Shan et al. 2016, Yadav et al. 2017, Bagert et al. 2021, Lowe et al. 2021, Lemon et al. 2022, Zhang et al. 2023, Ohkuni et al. 2025, Sad et al. 2025). For instance, in a humanized budding yeast library, histone oncomutants are unbiasedly screened for their effects on chromatin remodeling (Bagert et al. 2021). In the fission yeast *Schizosaccharomyces pombe*, H3K9M traps histone methyltransferase Clr4 and blocks H3K9 methylation *in trans* (Shan et al. 2016). Another oncohistone, H3G34R, reduces H3K36me3 levels and leads to HR repair defect and genomic instability in *S. pombe* (Yadav et al. 2017). Although H3K36me3 levels are also reduced in H3G34V, H3G34R displays more severe genomic instability than H3G34V does, probably due to more H3K27 methylation and a distinct profile of gene expression in H3G34R. However, H3G34W and nononcohistones H3G34M/Q (glycine-to-methionine/glutamine) fail to decrease H3K36me3 levels and thus exhibit no defects in DNA damage repair and genome stability (Lowe et al. 2021). In the budding yeast *Saccharomyces cerevisiae*, H3G34R/V/W/L exhibit similar sensitivities to caffeine, formamide, and HU, whereas H3K36M/R are more sensitive to cellular stress, such as caffeine. This is linked to their reduced extent of H3K36me3 levels (Lemon et al. 2022). Another study demonstrated that H3E50K/R (glutamic acid-to-lysine/arginine) confer greater sensitivities to caffeine and bleomycin than H3E50A (glutamic acid-to-alanine) does in *S. cerevisiae*, but they cause similar reductions in certain H3 N-tail PTMs (Sad et al. 2025). Moreover, the highly frequent H3E97K and uncommon H3E97A exhibit similar chromosomal instability by reducing the interaction with H4 and facilitating CENP-A mislocalization in *S. cerevisiae* (Ohkuni et al. 2025). In addition to oncohistone H3, yeast is also emerging as an excellent model for studying oncohistone H2B (Wan and Chan 2021). H2BE76K is the most frequent H2B oncomutant, and its conserved H2BE79K in *S. cerevisiae* is more sensitive to a high temperature than H2BE79Q (glutamic acid-to-glutamine), possibly because H2BE79K renders the nucleosome more unstable than H2BE79Q (Bennett et al. 2019).

Our previous study characterized the conserved and common H2B oncohistone mutants in *S. pombe*, whose genome contains a single histone H2B-encoding gene compared with two genes in *S. cerevisiae* and 23 genes in humans (Zhang et al. 2023). We revealed that H2BE112K and H2BE34K mutants have more severe genotoxic defects than those of H2BE112Q and H2BE34D, respectively. Importantly, H2BG52D (glycine-to-aspartic acid), H2BD67N (aspartic acid-to-asparagine), and H2BP102L (proline-to-leucine) reduce the levels of H2B monoubiquitination at Lys119 (H2B^ub^), leading to oncogenic phenotypes such as genomic instability by compromising the ability of HR repair (Qin et al. 2024). In addition to regulating HR, H2B^ub^ also modulates gene expression. Therefore, it remains unclear whether other amino acid substitutions at the H2B-Gly52/Asp67/Pro102 sites reduce H2B^ub^ levels and whether they cause genomic instability by altering gene expression. In this study, we constructed various oncomutants at a single site of the H2B gene *htb1-Gly52/Asp67/Pro102* in *S. pombe* and investigated their effects on genotoxic response, H2B^ub^ levels, and gene expression. Furthermore, we studied the effects of H2B^ub^ restoration on gene expression in *htb1-G52D, htb1-D67N*, and *htb1-P102L* oncomutants. Interestingly, among the various *htb1-Gly52/Asp67/Pro102* mutants, *htb1-G52D, htb1-D67N*, and *htb1-P102L* exclusively displayed significant genotoxic sensitivity, reduced H2B^ub^ levels, and altered gene expression. These effects were restored to normal levels after rescuing H2B^ub^ levels with the deletion of the H2B deubiquitinase *ubp8^+^*.

## Materials and methods

### *Schizosaccharomyces pombe* strains, plasmids, and antibodies

*Schizosaccharomyces pombe* strains were constructed by standard Polymerase Chain Reaction (PCR)-based transformation or mating (Bähler et al. 1998, Forsburg and Rhind 2006, Sabatinos and Forsburg 2010). Briefly, the DNA fragment containing 5′ UTR and upstream sequences (422 bp), CDS (381 bp), and 3′ UTR (639 bp) of the *htb1*^+^ gene was PCR amplified from *S. pombe* genomic DNA as 5′ overhang. The fragment of 3′ UTR downstream sequence (343 bp) of *htb1*^+^ was also PCR amplified as 3′ overhang. The 5′ overhang of *htb1*^+^ was cloned into the upstream of *kanMX6* at the *Bam*HI and *Bgl*II sites of the pFA6a-kanMX6 plasmid, and the 3′ overhang of *htb1*^+^ was cloned into the downstream of *kanMX6* at the *Sac*I and *Sac*II sites of pFA6a-kanMX6. The resulting plasmid was named as pGF2. Various *htb1-Gly52/Asp67/Pro102* (the number of an H2B residue in *S. pombe* is one less than that in humans) mutations in pGF2 were generated by site-directed mutagenesis. The 5′ overhang fragment with *htb1-Gly52/Asp67/Pro102* mutations, *kanMX6*, and the 3′ overhang of *htb1*^+^ were then PCR amplified to form the full fragment (3231 bp), which was transformed to *S. pombe* cells. The G418 resistant colonies were verified by colony PCR and sequenced for various *htb1-Gly52/Asp67/Pro102* mutations. The strain genotypes are listed in Table S1. The information on the plasmids is presented in Table S2. The use of antibodies is shown in Table S3.

### Spot assay of *S. pombe* growth

The spot assay was performed according to our previous publications (Feng et al. 2019, Qin et al. 2024, Lu et al. 2025). *Schizosaccharomyces pombe* cells were grown to the log phase, adjusted to an optical density (OD_600_) of 0.5, and serially 5-fold diluted. Each dilution was spotted onto plates containing the indicated genotoxic drugs at 30°C. The drugs used are hydroxyurea (HU) (Sigma, H8627, St. Louis, MO, USA), camptothecin (CPT) (Sigma, C9911), methyl methanesulfonate (MMS) (Sigma, 129925), phleomycin (Phleo) (MCE, HY-126490, Monmouth Junction, NJ, USA), bleomycin (Bleo) (MCE, HY-17565), and 6-azauracil (6-AU) (Sigma, A1757). The plates were incubated for the indicated days and photographed.

### Protein extraction and immunoblotting

Whole-cell protein extraction and immunoblotting were based on our previous methods (Feng et al. 2019, Qin et al. 2024, Lu et al. 2025). The log phase *S. pombe* cells with 10 ml at OD_600_ = 1.0 were incubated in lysis buffer (0.9 M NaOH and 3.5% 2-mercaptoethanol) and then added with an equal volume of 55% trichloroacetic acid (Sigma, 91228). The cells were then centrifuged and resuspended in 2x protein SDS-loading buffer. After neutralization with 1 M Tris, the cells were boiled at 90°C for 5 min. The proteins were subsequently subjected to SDS-PAGE, transferred, and detected with an Odyssey infrared imaging system (Li-COR) (Lincoln, NE, USA). After exposure, the blots containing whole-cell proteins were stained with Coomassie Brilliant Blue (CBB), which served as an independent loading control of total proteins. The quantitation of the abundance of H2B^ub^ or FLAG-tagged protein is normalized to total protein levels measured by CBB staining (Leng et al. 2022).

### RNA-seq analysis of the transcriptome

RNA-seq was performed based on our previous studies (Qin et al. 2024, Lu et al. 2025). Total RNA for RNA-seq was extracted with a Trizol Kit (Invitrogen, Carlsbad, CA, USA) and mRNA was enriched with oligo(dT) beads. Following fragmentation, cDNA was synthesized, ligated, and amplified via PCR. The PCR products were sequenced with an Illumina HiSeq 2500 instrument by Gene Denovo Biotechnology. The raw reads were filtered and removed from rRNA. Clean reads were mapped to the *S. pombe* reference genome (Ensembl_release45) using HISAT2. The read count was transformed to the fragment per kilobase of transcript per million mapped reads value as a measure of gene expression levels via StringTie. The differentially expressed genes (DEGs) were defined as transcripts with false discovery rate or *q* value below 0.05 and more than 2-fold change in expression. The data were averaged from three independent biological repeats and analyzed with the Omicsmart online platform (http://www.omicsmart.com). The expression of all genes in the indicated strains is listed in Tables S5–S7. A list of DEGs in the indicated strains is included in Tables S8–S10.

### RNA extraction

Total RNA was prepared as described previously with some modifications (Feng et al. 2013). Briefly, 50 ml of cells at OD_600_ = 1.0 were harvested and resuspended in 750 µl of lysis buffer (50 mM NaOAc, 10 mM EDTA, and 1% SDS). The resuspended cells were combined with 750 µl of acid phenol (pH 4.7) (Ambion, Austin, TX, USA) and incubated at 65°C for 1 h with shaking. The cell lysates were cooled and centrifuged for 5 min at 20 000 × *g*. The aqueous phase was extracted with phenol–chloroform (pH 7.5) repeatedly until the interfaces were no longer white. After the addition of 3 M NaOAc and 100% ethanol, the RNA was precipitated and stored at −80°C until use.

### Reverse transcription and quantitative PCR

The reverse transcription and quantitative PCR (RT-qPCR) assay was conducted and analyzed as described in our previous publication (Feng et al. 2013). Total RNA (200 ng µl^−1^) was treated with gDNA Wiper Mix (Vazyme, R323, Nanjing, Jiangsu, China) at 42°C for 2 min to remove any contaminated genomic DNA. Then, 500 ng of total RNA was reverse transcribed into cDNA using random primers with the HiScript Ⅲ qRT Super Mix (Vazyme, R323). After cDNA was synthesized and diluted to ~10 ng ml^−1^, 6 µl of this cDNA template, 10 µl of 2x SYBR Green qPCR master mix (Vazyme, Q712), and 4 µl of primer sets for the indicated genes were mixed and run on an ABI QuantStudio Real-Time PCR instrument (Carlsbad, CA, USA). The expression of the genes was determined by the comparative Ct (2^−Ct^) method and normalized to that of *act1*^+^, whose expression remained unchanged under the indicated experimental conditions in RNA-seq and immunoblotting assays. We further normalized the gene expression in the indicated mutants to that in the wild-type (WT) (arbitrarily set as 1) as the relative fold change, which was averaged from three independent biological repeats. The RT-qPCR primers used are listed in Table S4.

### Chromatin immunoprecipitation and quantitative PCR

The chromatin immunoprecipitation and quantitative PCR (ChIP-qPCR) assay was performed as previously described (Feng et al. 2019, Qin et al. 2024). To prevent H2B^ub^ from deubiquitination, *S. pombe* cells grown in YES media were treated with 20 mM of the fresh deubiquitinase inhibitor *N*-ethylmaleimide (NEM) (Sigma, E1271) for 30 min before cross-linking. The cells with 100 ml at OD_600_ = 1.0 were then cross-linked with 1% formaldehyde (Sigma, 47608) at 25°C for 20 min and neutralized by adding a 1/20 volume of 2.5 M glycine. The cells were subsequently resuspended in lysis buffer [50 mM HEPES, pH 7.2; 150 mM NaCl; 1 mM EDTA; 1% Triton X-100; 0.1% sodium deoxycholate (SDC)] supplemented with 1x yeast protease inhibitors (Sangon, C510026, Shanghai, China) and/or 20 mM of fresh NEM. The cells were then bead-beaten with FastPrep (MP Biomedicals, CA, USA). After centrifugation, the supernatant was sonicated to an average of 500 bp of DNA fragments as input. The chromatin samples were incubated with the indicated antibodies and then incubated with protein A/G agarose beads (Thermo, 26 159, Waltham, MA, USA). After washing with lysis buffer, high-salt buffer (50 mM HEPES, pH 7.2; 500 mM NaCl; 1 mM EDTA; 1% Triton X-100; 0.1% SDC), and LiCl buffer (20 mM Tris, pH 8.0; 250 mM LiCl; 0.5% NP-40; 0.5% SDC), the immunoprecipitated chromatin was eluted and reverse cross-linked with 1% SDS at 65°C overnight. The immunoprecipitated and input DNA were digested with 100 µg ml^−1^ proteinase K (NEB, P8102, Ipswich, MA, USA) and 100 µg ml^−1^ RNase A (Thermo, EN0531, Waltham, MA, USA) and purified with the MinElute PCR purification kit (Qiagen, 28004, Düsseldorf, Germany). 6 µl of diluted DNA template, 10 µl of 2x SYBR Green PCR master mix (Vazyme, Q712), and 4 µl of the indicated primers (Table S4) were mixed and run on an ABI QuantStudio Real-Time PCR instrument. The enrichment was calculated as the percentage of immunoprecipitated DNA relative to the input DNA levels by the comparative Ct (2^−Ct^) method. We normalized the enrichment in the H2B oncomutants to that in the WT (set as 1) as the relative fold change at the indicated locus.

### Structural prediction of nucleosomes with oncohistones

Structural predictions of *S. pombe* nucleosomes with oncohistones were conducted via the AlphaFold 3 server (https://www.alphafoldserver.com) with default parameters (Jumper et al. 2021, Abramson et al. 2024). The structures were visualized via PyMOL software.

### Statistical analysis

Statistical analysis was conducted using GraphPad Prism software (San Diego, CA, USA). All the data are expressed as the means with error bars indicating standard deviations (SDs) from two or three independent biological repeats. ns, *, **, ***, and ^****^ indicate no significance, *P* < .05, *P* < .01, *P* < .001, and *P* < .0001, respectively.

## Results

### The diverse *htb1-Gly52* oncomutants exhibit distinct temperature and genotoxic phenotypes

**Figure 1.**
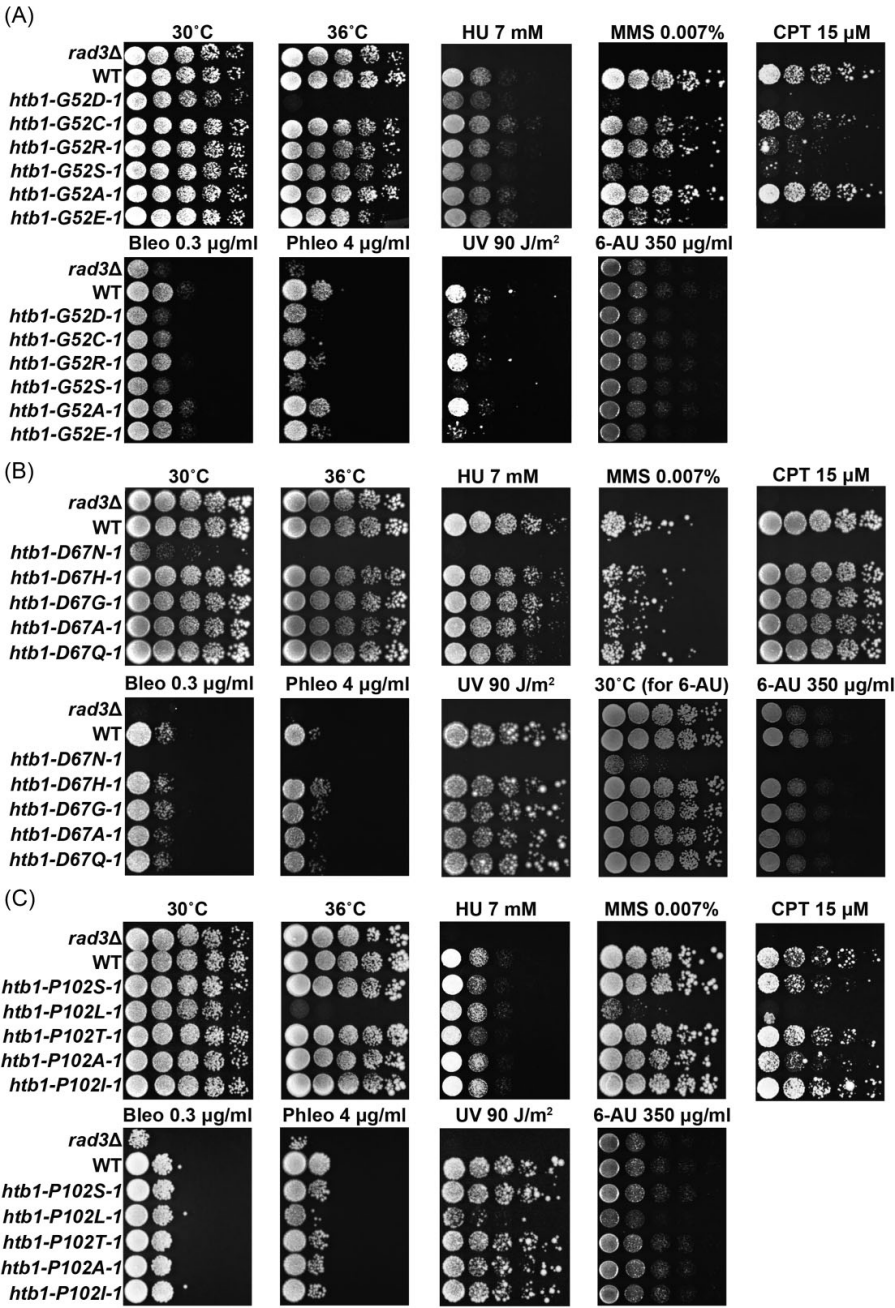
The temperature and genotoxic phenotypes of the *htb1-Gly52*/*Asp67*/*Pro102* diverse mutants. (A) The growth of WT (TK8), *htb1-G52D* (YGF277), *htb1-G52C* (YGF507), *htb1-G52R* (YGF510), *htb1-G52S* (YGF460), *htb1-G52A* (YGF459), and *htb1-G52E* (YGF508) strains under indicated conditions. (B) The growth of WT (TK8), *htb1-D67N* (YGF324), *htb1-D67H* (YGF515), *htb1-D67G* (YGF514), *htb1-D67A* (YGF513), and *htb1-D67Q* (YGF509) strains under indicated conditions. (C) The growth of WT (TK8), *htb1-P102S* (YGF325), *htb1-P102L* (YGF279), *htb1-P102T* (YGF512), *htb1-P102A* (YGF511), and *htb1-P102I* (YGF505) strains under the indicated conditions. The *rad3*Δ (LD297) strain is a positive control. The length of incubation time is 4 days.

In a previous study, we modeled H2B oncohistones by mutating their single gene *htb1*^+^ and expressing each variant as the sole copy of histone H2B in *S. pombe*. We found that the *htb1-G52D, htb1-D67N*, and *htb1-P102L* oncomutations cause the HR repair defect and genomic instability by reducing H2B^ub^ levels (Qin et al. 2024). To test whether other amino acid substitutions at the H2B-Gly52 residue have similar effects on genome maintenance, we created four other different missense oncomutants at the same H2B-Gly52 residue in *S. pombe*. These mutants were ranked by their mutation frequencies in human tumors as follows: *htb1-G52D* (glycine-to-aspartic acid) > *htb1-G52C* (glycine-to-cysteine) > *htb1-G52R* (glycine-to-arginine) > *htb1-G52S* (glycine-to-serine) > *htb1-G52A* (glycine-to-alanine) (Bennett et al. 2019, Nacev et al. 2019). Additionally, we introduced a negatively charged Glu to generate the nononcohistone *htb1-G52E*, which is similar to *htb1-G52D* but has not been reported in tumors. We subsequently evaluated their growth by spotting two independent repeats of strains onto plates under the following conditions: optimal temperature (30°C); high temperature (36°C); replication stress (HU, MMS, and CPT); DNA damage (bleomycin, phleomycin, and UV irradiation); and transcription elongation inhibitor (6-AU). Among the *htb1-Gly52* mutants, only *htb1-G52D* exhibited slightly slower growth compared with WT and other *htb1-Gly52* mutants at 30°C. Moreover, *htb1-G52D* was temperature-sensitive at 36°C, whereas *htb1-G52E* exhibited mild sensitivity (Fig. 1A and Fig. S1A). Interestingly, all the mutants except *htb1-G52A* showed varying degrees of sensitivity to at least one genotoxic agent (Fig. 1A and Fig. S1A). This implies that these five H2B mutants (*htb1-G52D, htb1-G52C, htb1-G52R, htb1-G52S*, and *htb1-G52E*) possibly exhibit DNA damage repair defects and genomic instability. In particular, the *htb1-G52D* mutant showed the most severe sensitivity, whereas the *htb1-G52E* and *htb1-G52S* showed less sensitivity than that of *htb1-G52D*. The genotoxic sensitivities of *htb1-G52C* and *htb1-G52R* were significantly lower compared with those of *htb1-G52D, htb1-G52E*, and *htb1-G52S* (Fig. 1A and Fig. S1A). When exposed to 6-AU, the *htb1-G52D* exhibited little sensitivity, while *htb1-G52E* displayed sensitivity (Fig. 1A and Fig. S1A). As *htb1-G52D* or its analogue *htb1-G52E* possibly reduces H2B^ub^ levels (Qin et al. 2024), this 6-AU finding is consistent with a key role of H2B^ub^ in transcription regulation and suggests that the other four *htb1-Gly52* mutants potentially have no effects on H2B^ub^ levels.

### Only *htb1-D67N* among the *htb1-Asp67* diverse oncomutants is sensitive to high temperature and DNA damage

In addition, we constructed three *htb1-Asp67* oncomutants and ranked them according to their frequencies in human tumors as follows: *htb1-D67N* >* htb1-D67H* (aspartic acid-to-histidine) > *htb1-D67G* (aspartic acid-to-glycine). The nononcohistone *htb1-D67A* (aspartic acid-to-alanine) was also generated as an expected negative control. Moreover, Gln was incorporated to generate the *htb1-D67Q* (aspartic acid-to-glutamine) mutant, which shares similar amino acid properties with *htb1-D67N*. We then investigated the effects of high temperature, DNA damage, and replication stress on their growth. We observed that only *htb1-D67N* showed a growth defect at the optimal temperature of 30°C and displayed a more severe defect at 36°C (Fig. 1B and Fig. S1B). Surprisingly, only the *htb1-D67N* exhibited sensitivities to replication stress (HU, MMS, and CPT treatments) and DNA damage (bleomycin, phleomycin, and UV irradiation) (Fig. 1B and Fig. S1B). As expected, the *htb1-D67N*, which reduces H2B^ub^ levels (Qin et al. 2024), showed strong sensitivity in the presence of 6-AU treatment (Fig. 1B and Fig. S1B), suggesting that the other four *htb1-Asp67* mutants potentially display no defect in H2B^ub^ levels.

### Only *htb1-P102L* among the *htb1-Pro102* diverse oncomutants shows the sensitivities to high temperature and genotoxins

Moreover, we constructed three different *htb1-Pro102* oncomutants and ranked them by mutation frequency as follows: *htb1-P102S* (proline-to-serine) > *htb1-P102L* > *htb1-P102T* (proline-to-threonine). The *htb1-P102A* (proline-to-alanine) was created as a nononcohistone control, and Ile was used to generate the *htb1-P102I* (proline-to-isoleucine), which resembles *htb1-P102L*. We observed that all the *htb1-Pro102* mutants were not defective in growth at optimal temperature (30°C). Interestingly, only the *htb1-P102L* grew much more slowly at 36°C (Fig. 1C and Fig. S1C). Similarly, only the *htb1-P102L* exhibited the great sensitivity to MMS and CPT but not HU, and the mild sensitivity to DNA damage (bleomycin, phleomycin, and UV irradiation). The other four *htb1-Pro102* mutants showed no such sensitivities (Fig. 1C and Fig. S1C). In the case of 6-AU, only the *htb1-P102L*, which reduces H2B^ub^ levels (Qin et al. 2024), exhibited sensitivity (Fig. 1C and Fig. S1C), suggesting that the other four *htb1-Pro102* mutants do not potentially affect H2B^ub^ levels.

### The *htb1-G52D, htb1-D67N*, and *htb1-P102L* among the *htb1-Gly52/Asp67/Pro102* diverse oncomutants decrease H2B^ub^ levels

**Figure 2.**
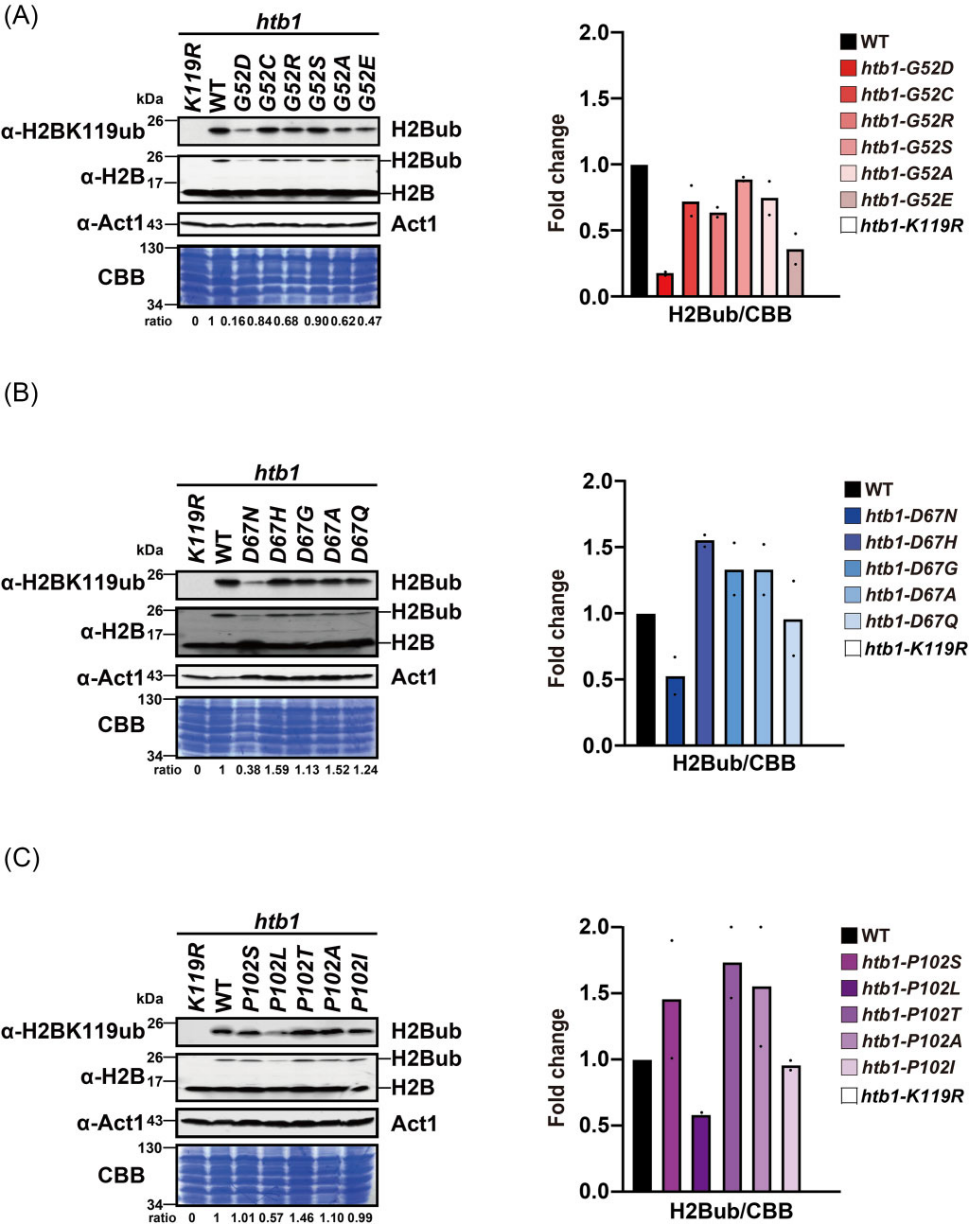
The levels of H2B^ub^ in the *htb1-Gly52*/*Asp67*/*Pro102* diverse mutants. (A) Immunoblots of H2B^ub^ levels in *htb1-K119R* (YGF226), WT (TK8), *htb1-G52D* (YGF277), *htb1-G52C* (YGF507), *htb1-G52R* (YGF510), *htb1-G52S* (YGF460), *htb1-G52A* (YGF459), and *htb1-G52E* (YGF508) cells. (B) Immunoblots of H2B^ub^ amounts in *htb1-K119R* (YGF226), WT (TK8), *htb1-D67N* (YGF324), *htb1-D67H* (YGF515), *htb1-D67G* (YGF514), *htb1-D67A* (YGF513), and *htb1-D67Q* (YGF509) cells. (C) Immunoblots of H2B^ub^ abundance in *htb1-K119R* (YGF226), WT (TK8), *htb1-P102S* (YGF325), *htb1-P102L* (YGF279), *htb1-P102T* (YGF512), *htb1-P102A* (YGF511), and *htb1-P102I* (YGF505) cells. Left panel: the representative immunoblots from a biological replicate are shown. The intensity of the H2B^ub^ band is normalized to that of CBB staining. The fold change of this normalization factor in the indicated mutants relative to WT (set as 1) is denoted as the ratio at the bottom. Right panel: quantification of the relative fold change is plotted. Data represent the mean with minimum and maximum values from two independent biological replicates.

To investigate the effects of diverse *htb1-Gly52/Asp67/Pro102* mutants on H2B^ub^ levels, we performed an immunoblotting assay to measure H2B^ub^ levels. The *htb1-K119R* mutant served as a control for the absence of H2B^ub^. Among the six *htb1-Gly52* mutants, the H2B^ub^ levels were significantly decreased in *htb1-G52D* and *htb1-G52E* cells (Fig. 2A and Fig. S2A). We also found that the H2B^ub^ levels in *htb1-D67N* alone were reduced among the five *htb1-Asp67* mutants (Fig. 2B and Fig. S2B). Moreover, only *htb1-P102L* expressed lower levels of H2B^ub^ compared with the other four *htb1-Pro102* mutants (Fig. 2C and Fig. S2C). The levels of H2B were fairly similar among these H2B variants. Collectively, the certain *htb1-Gly52/Asp67/Pro102* mutants with decreased H2B^ub^ levels also show genotoxic sensitivity. This strong correlation indicates that H2B^ub^ plays a crucial role in the response of *htb1-Gly52/Asp67/Pro102* oncomutants to DNA damage.

### The *htb1-G52D, htb1-D67N*, and *htb1-P102L* among *htb1-Gly52/Asp67/Pro102* diverse oncomutants alter genome-wide gene expression

**Figure 3.**
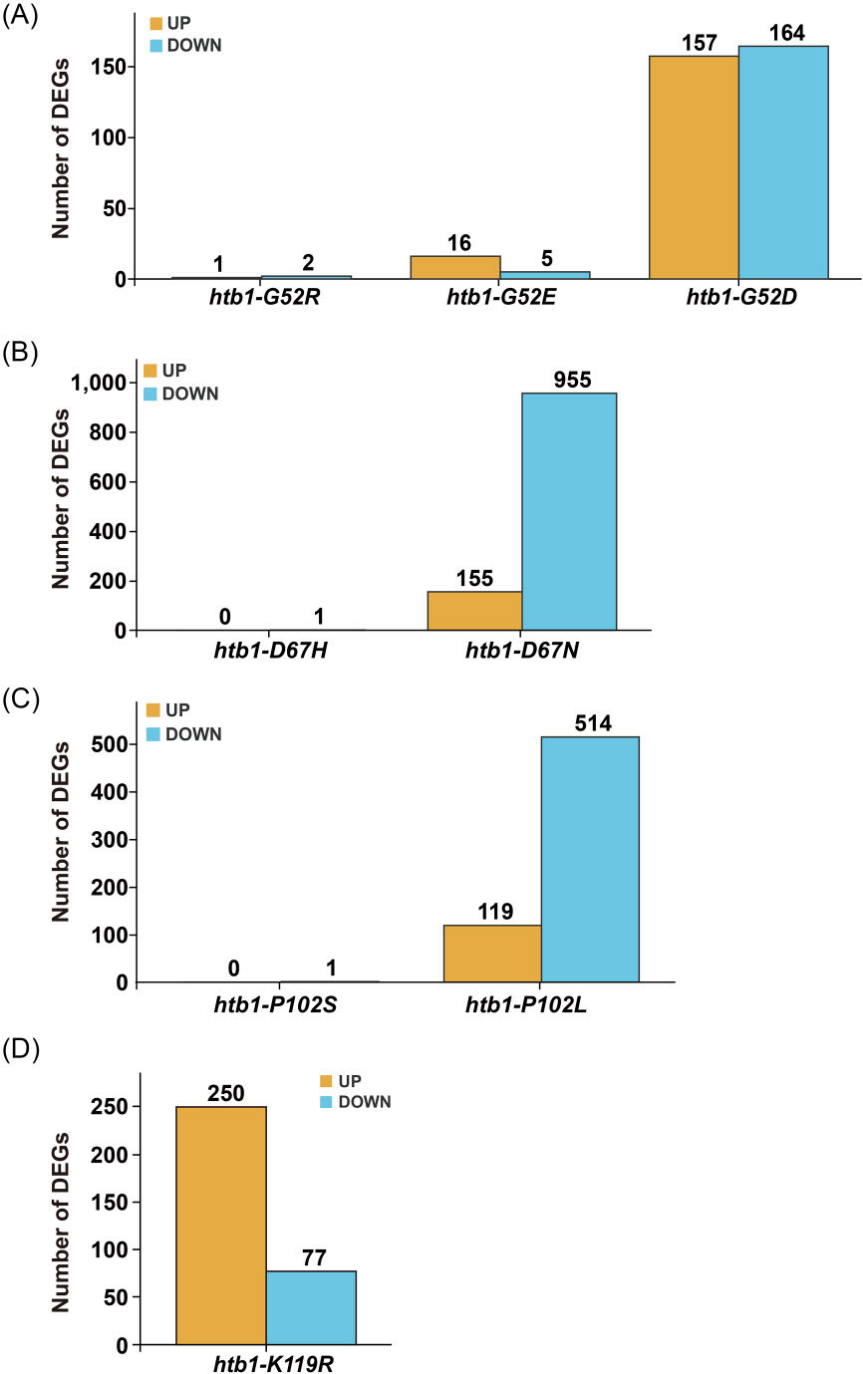
RNA-seq analysis of gene expression in the indicated *htb1-Gly52*/*Asp67*/*Pro102* oncomutants. (A) RNA-seq analysis of DEGs relative to WT cells in *htb1-G52D* (YGF277), *htb1-G52R* (YGF510), and *htb1-G52E* (YGF508). (B) RNA-seq analysis of DEGs relative to WT cells in *htb1-D67N* (YGF324) and *htb1-D67H* (YGF515). (C) RNA-seq analysis of DEGs relative to WT cells in *htb1-P102S* (YGF325) and *htb1-P102L* (YGF279). (D) RNA-seq analysis of DEGs relative to WT cells in *htb1-K119R* (YGF226). The number of genes with up- and downregulated expression is indicated at the top of the column.

Our previous finding showed that a reduction in H2B^ub^ levels impairs Rad51 recruitment for HR repair in *htb1-G52D* and *htb1-P102L* cells (Qin et al. 2024). In addition to its role in DNA repair, H2B^ub^ plays a key role in regulating transcription (Xiao et al. 2005, Tanny et al. 2007, Fuchs and Oren 2014, Chen et al. 2022). We thus hypothesized that the reduction in H2B^ub^ levels could lead to DNA repair defects in *htb1-Gly52/Asp67/Pro102* mutants by affecting the transcriptome. To test this hypothesis, we conducted an RNA-seq experiment and analyzed the transcriptomes of *htb1-G52D/E/R, htb1-D67N/H*, and *htb1-P102L/S* as a proof-of-principle. We found that the expression of 157 genes was significantly upregulated and 164 genes were downregulated in *htb1-G52D*, whereas the expression of few genes was altered in *htb1-G52E/R* (Fig. 3A and Table S8). Moreover, the expression of 155 genes was elevated, and the expression of 955 genes was reduced in *htb1-D67N*. In contrast, the expression of only one gene was altered in *htb1-D67H* (Fig. 3B and Table S8). In the case of *htb1-P102L*, the expression of 119 genes was increased, and the expression of 514 genes was decreased, whereas the expression of only one gene was decreased in *htb1-P102S* (Fig. 3C and Table S8). Moreover, the Venn Diagram demonstrated the remarkable difference in up- and downregulated genes in *htb1-G52D* (Fig. S3A), *htb1-D67N* (Fig. S3B), and *htb1-P102L* (Fig. S3C), compared with *htb1-G52E/R, htb1-D67H*, and *htb1-P102S*, respectively. Gene ontology (GO) analysis also revealed different numbers and classifications of altered genes in *htb1-G52D* (Fig. S3D), *htb1-D67N* (Fig. S3E), and *htb1-P102L* (Fig. S3F). To further test whether altered gene expression in *htb1-G52D*/*D67N/P102L* is associated with H2B^ub^ levels, we performed RNA-seq in *htb1-K119R* and found that the expression of 250 genes was significantly increased and that the expression of 77 genes was decreased (Fig. 3D and Table S9). We noticed that there were a significant number of overlapping genes with up- and downregulated expression between *htb1-K119R* and *htb1-G52D* (Fig. S3G), *htb1-D67N* (Fig. S3H), and *htb1-P102L* (Fig. S3I). We also observed similar GO patterns of altered genes between *htb1-K119R* and *htb1-G52D*/*D67N/P102L* (Fig. S3J). These results suggest that altered gene expression in *htb1-G52D*/*D67N/P102L* correlates with the genes regulated by H2B^ub^.

### The downregulated transcripts and proteins in *htb1-G52D, htb1-D67N*, and *htb1-P102L* are correlated with reduced H2B^ub^ levels

**Figure 4.**
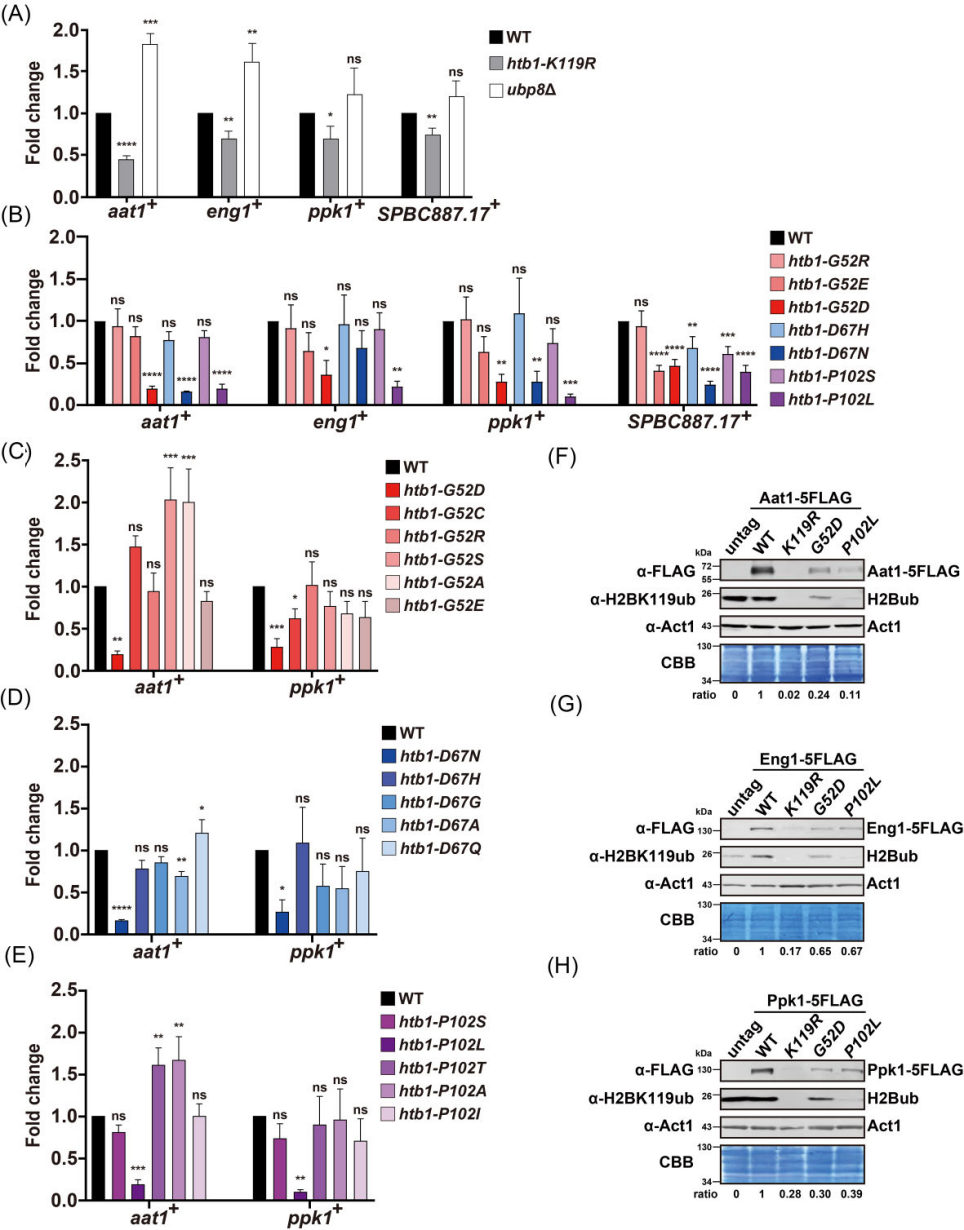
RT-qPCR and immunoblotting analysis of gene expression in the *htb1-Gly52*/*Asp67*/*Pro102* various mutants. (A) RT-qPCR analysis of *aat1*^+^, *eng1*^+^, *ppk1*^+^, and *SPBC887.17*^+^ transcripts in *htb1-K119R* (YGF226) and *ubp8*Δ (YGF415) mutants. (B) RT-qPCR validation of *aat1*^+^, *eng1*^+^, *ppk1*^+^, and *SPBC887.17*^+^ transcripts in the indicated and RNA-seq analyzed *htb1-Gly52/Asp67/Pro102* oncomutants. (C) RT-qPCR analysis of *aat1*^+^ and *ppk1*^+^ transcripts in *htb1-G52D* (YGF277), *htb1-G52C* (YGF507), *htb1-G52R* (YGF510), *htb1-G52S* (YGF460), *htb1-G52A* (YGF459), and *htb1-G52E* (YGF508) cells. (D) RT-qPCR analysis of *aat1*^+^ and *ppk1*^+^ transcripts in *htb1-D67N* (YGF324), *htb1-D67H* (YGF515), *htb1-D67G* (YGF514), *htb1-D67A* (YGF513), and *htb1-D67Q* (YGF509) cells. (E) RT-qPCR analysis of *aat1*^+^ and *ppk1*^+^ transcripts in *htb1-P102S* (YGF325), *htb1-P102L* (YGF279), *htb1-P102T* (YGF512), *htb1-P102A* (YGF511), and *htb1-P102I* (YGF505) cells. The fold change of gene expression in the indicated cells relative to that of WT (set as 1) is shown as mean ± SD (*n* = 3). A one-way analysis of variance (ANOVA) is performed to compare multiple data sets to WT. (F) Immunoblots of Aat1-5FLAG protein levels in untag (TK8), WT (YGF644), *htb1-K119R* (YGF645), *htb1-G52D* (YGF646), and *htb1-P102L* (YGF647) cells. (G) Immunoblots of Eng1-5FLAG protein levels in untag (TK8), WT (YGF632), *htb1-K119R* (YGF633), *htb1-G52D* (YGF634), and *htb1-P102L* (YGF635) cells. (H) Immunoblots of Ppk1-5FLAG protein levels in untag (TK8), WT (YGF591), *htb1-K119R* (YGF592), *htb1-G52D* (YGF594), and *htb1-P102L* (YGF595) cells. The intensity of the FLAG-tagged protein band is normalized to that of CBB staining. The fold change of this normalization factor in the indicated mutants relative to WT (set as 1) is depicted as the ratio at the bottom.

We previously found that the expression of DNA repair genes was not generally affected in *htb1-G52D*/*D67N/P102L* (Qin et al. 2024). Thus, to further validate that H2B^ub^ is involved in gene dysregulation in *htb1-G52D*/*D67N/P102L*, we focused on the expression of four representative genes: *aat1*^+^, *eng1*^+^, *ppk1*^+^, and *SPBC887.17*^+^, whose transcript levels were downregulated in *htb1-K119R* according to previous microarray data (Tanny et al. 2007) and our RNA-seq data (Fig. 3D and Table S9). The *aat1*^+^ gene encodes an amino acid transmembrane transporter in the plasma membrane. The *eng1*^+^ gene produces an endoglucanase involved in cell wall catabolism. The *ppk1*^+^ gene encodes the serine/threonine protein kinase Ppk1, and its *S. cerevisiae* homolog Kin4 is a spindle position checkpoint and mitosis regulator (D’Aquino et al. 2005, Pereira and Schiebel 2005, Ekal et al. 2023). The *SPBC887.17*^+^ gene is inferred to encode a guanine and adenine transmembrane transporter in the plasma membrane. We performed RT-qPCR experiments and validated that the levels of their transcripts were indeed decreased in *htb1-K119R* (Fig. 4A). We also found that their transcript levels were increased in *ubp8*Δ, which is a null mutant of H2B deubiquitinase *ubp8*^+^ (Fig. 4A). The RNA levels of *aro1*^+^ gene, which is independent of H2B^ub^ regulation and used as a negative control (Tanny et al. 2007), were consistently unaltered in our *htb1-K119R* and *ubp8*Δ mutants (Fig. S4A). These data suggest that transcription of these four genes is regulated by H2B^ub^. Subsequently, we used RT-qPCR to verify the RNA levels of these four genes in certain *htb1-Gly52/Asp67/Pro102* mutants that were subjected to RNA-seq. We found that the levels of *aat1*^+^, *eng1*^+^, *ppk1*^+^, and *SPBC887.17*^+^ transcripts were reduced in *htb1-G52D, htb1-D67N*, and *htb1-P102L* but not in *htb1-G52R/G52E, htb1-D67H*, and *htb1-P102S* (Fig. 4B). The RNA levels of the control gene *aro1*^+^ were unaltered in these mutants (Fig. S4B). Additionally, we determined the RNA levels of *aat1*^+^ and *ppk1*^+^ in all the constructed *htb1-Gly52/Asp67/Pro102* mutants. Consistent with the results of H2B^ub^ levels, *htb1-G52D* (Fig. 4C), *htb1-D67N* (Fig. 4D), and *htb1-P102L* (Fig. 4E) exclusively exhibited reduced RNA levels of *aat1*^+^ and *ppk1*^+^. To verify these results, we tagged *aat1*^+^, *eng1*^+^, and *ppk1*^+^ with 5x FLAG at their C-termini and performed immunoblotting to examine whether their protein levels were correlated with their transcript levels. Consistently, the abundance of the Aat1-5FLAG (Fig. 4F), Eng1-5FLAG (Fig. 4G), and Ppk1-5FLAG (Fig. 4H) proteins were decreased in *htb1-G52D, htb1-P102L*, and more significantly reduced in *htb1-K119R*. Taken together, the correlations in these representative RNA and protein levels between *htb1-K119R* and *htb1-G52D/D67N/P102L* suggest that gene dysregulation in *htb1-G52D/D67N/P102L* mutants is the consequence of compromised H2B^ub^ levels.

### The dysregulation of transcripts in *htb1-G52D, htb1-D67N*, and *htb1-P102L* is restored by the absence of *ubp8*^+^

**Figure 5.**
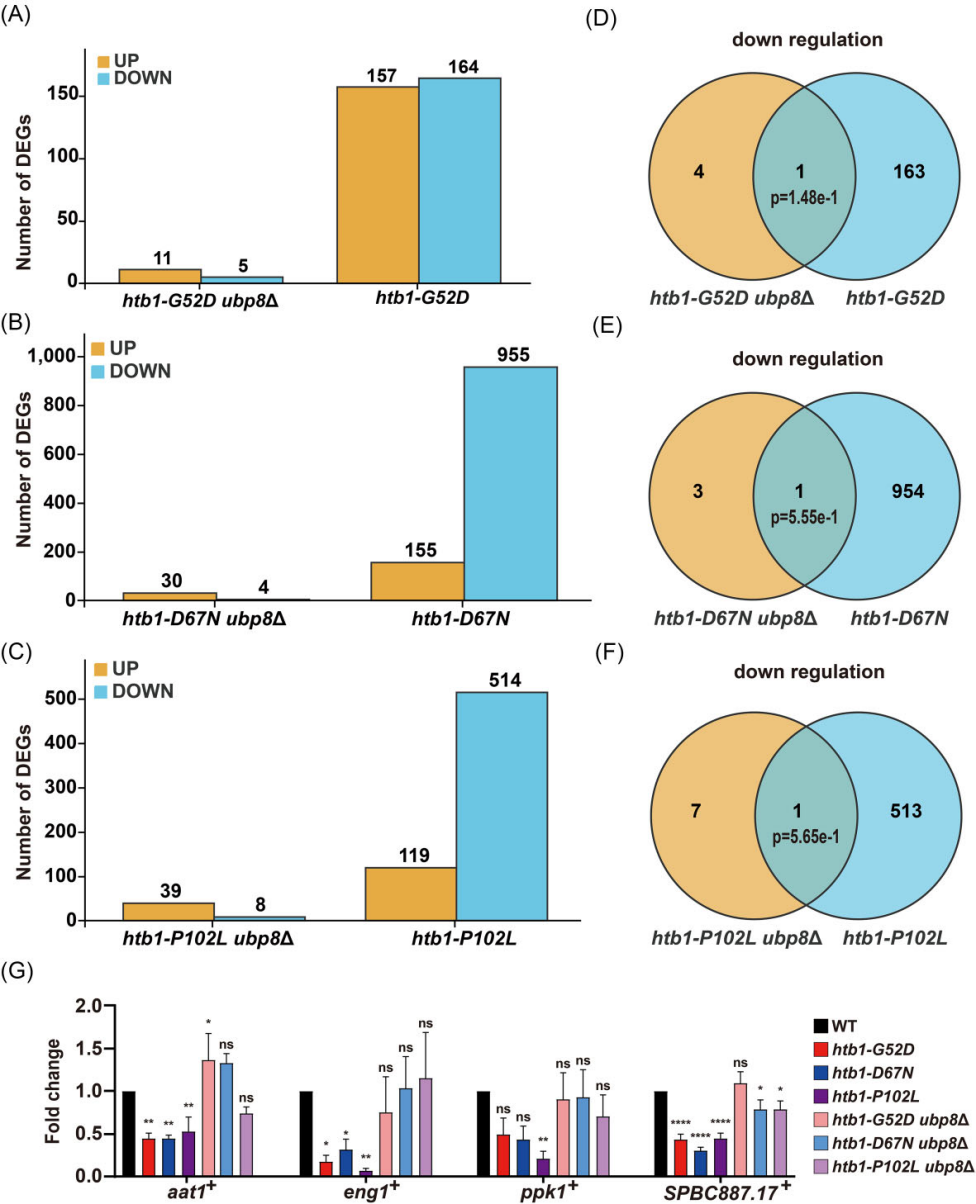
RNA-seq and RT-qPCR analysis of gene expression in the *htb1-G52D*/*D67N*/*P102L* oncomutants with *ubp8*Δ. (A) RNA-seq analysis of DEGs relative to WT cells in *htb1-G52D* (YGF277) and *htb1-G52D ubp8*Δ (YGF416) cells. (B) RNA-seq analysis of DEGs relative to WT cells in *htb1-D67N* (YGF324) and *htb1-D67N ubp8*Δ (YGF443) cells. (C) RNA-seq analysis of DEGs relative to WT cells in *htb1-P102L* (YGF279) and *htb1-P102L ubp8*Δ (YGF417) cells. The number of genes with up- and downregulated expression is indicated at the top of the column. (D) Venn Diagrams of downregulated genes in *htb1-G52D* (YGF277) and *htb1-G52D ubp8*Δ (YGF416). (E) Venn Diagrams of downregulated genes in *htb1-D67N* (YGF324) and *htb1-D67N ubp8*Δ (YGF443). (F) Venn Diagrams of downregulated genes in *htb1-P102L* (YGF279) and *htb1-P102L ubp8*Δ (YGF417). Fisher’s exact test is used to calculate the *P*-values of significant differences in Venn Diagrams. (G) RT-qPCR of *aat1*^+^, *eng1*^+^, *ppk1*^+^, and *SPBC887.17*^+^ transcripts in the *htb1-G52D, htb1-D67N*, and *htb1-P102L* oncomutants and their double mutants with *ubp8*Δ. The fold change of gene expression in the indicated cells relative to that of WT (set as 1) is shown as mean ± SD (*n* = 3). A one-way ANOVA is performed for multiple comparisons to WT.

To further support that H2B^ub^ deficiency in *htb1-G52D, htb1-D67N*, and *htb1-P102L* is the main mechanism for the dysregulation of their transcripts, we performed RNA-seq experiments in our previously constructed strains *htb1-G52D ubp8*Δ, *htb1-D67N ubp8*Δ, and *htb1-P102L ubp8*Δ, where deletion of *ubp8*^+^ restored H2B^ub^ levels (Qin et al. 2024). Interestingly, the number of altered transcripts, particularly for the reduced transcripts, in *htb1-G52D ubp8*Δ (Fig. 5A and Table S10), *htb1-D67N ubp8*Δ (Fig. 5B and Table S10), and *htb1-P102L ubp8*Δ (Fig. 5C and Table S10) was significantly lower than that in *htb1-G52D, htb1-D67N*, and *htb1-P102L*, respectively. The Venn Diagram also showed no significant overlap for the downregulated genes in *htb1-G52D ubp8*Δ (Fig. 5D), *htb1-D67N ubp8*Δ (Fig. 5E), and *htb1-P102L ubp8*Δ (Fig. 5F) compared with *htb1-G52D, htb1-D67N*, and *htb1-P102L*, respectively. Despite there being a significant overlap for the upregulated and dysregulated genes, the number of overlapping genes was much smaller in *htb1-G52D* (Fig. S5A), *htb1-D67N* (Fig. S5B), and *htb1-P102L* (Fig. S5C). Moreover, GO analysis revealed a significant difference in the number and classification of affected genes in *htb1-G52D ubp8*Δ (Fig. S5D), *htb1-D67N ubp8*Δ (Fig. S5E), and *htb1-P102L ubp8*Δ (Fig. S5F). To validate these RNA-seq data, we measured the levels of *aat1*^+^, *eng1*^+^, *ppk1*^+^, and *SPBC887.17*^+^ transcripts in *htb1-G52D, htb1-D67N*, and *htb1-P102L* with and without *ubp8*^+^ by RT-qPCR. Consistent with the result shown in Fig. 4(B), the levels of *aat1*^+^, *eng1*^+^, *ppk1*^+^, and *SPBC887.17*^+^ transcripts were reduced in *htb1-G52D, htb1-D67N*, and *htb1-P102L*. After deleting *ubp8*^+^, the levels of these transcripts were almost restored to WT levels (Fig. 5G). Collectively, these data indicate that the altered levels of transcripts in *htb1-G52D, htb1-D67N*, and *htb1-P102L* are mechanistically due to H2B^ub^ deficiency.

### The downregulated transcripts in *htb1-G52D, htb1-D67N*, and *htb1-P102L* are caused by the reduced H2B^ub^ levels and the defect in RNA polymerase II elongation

**Figure 6.**
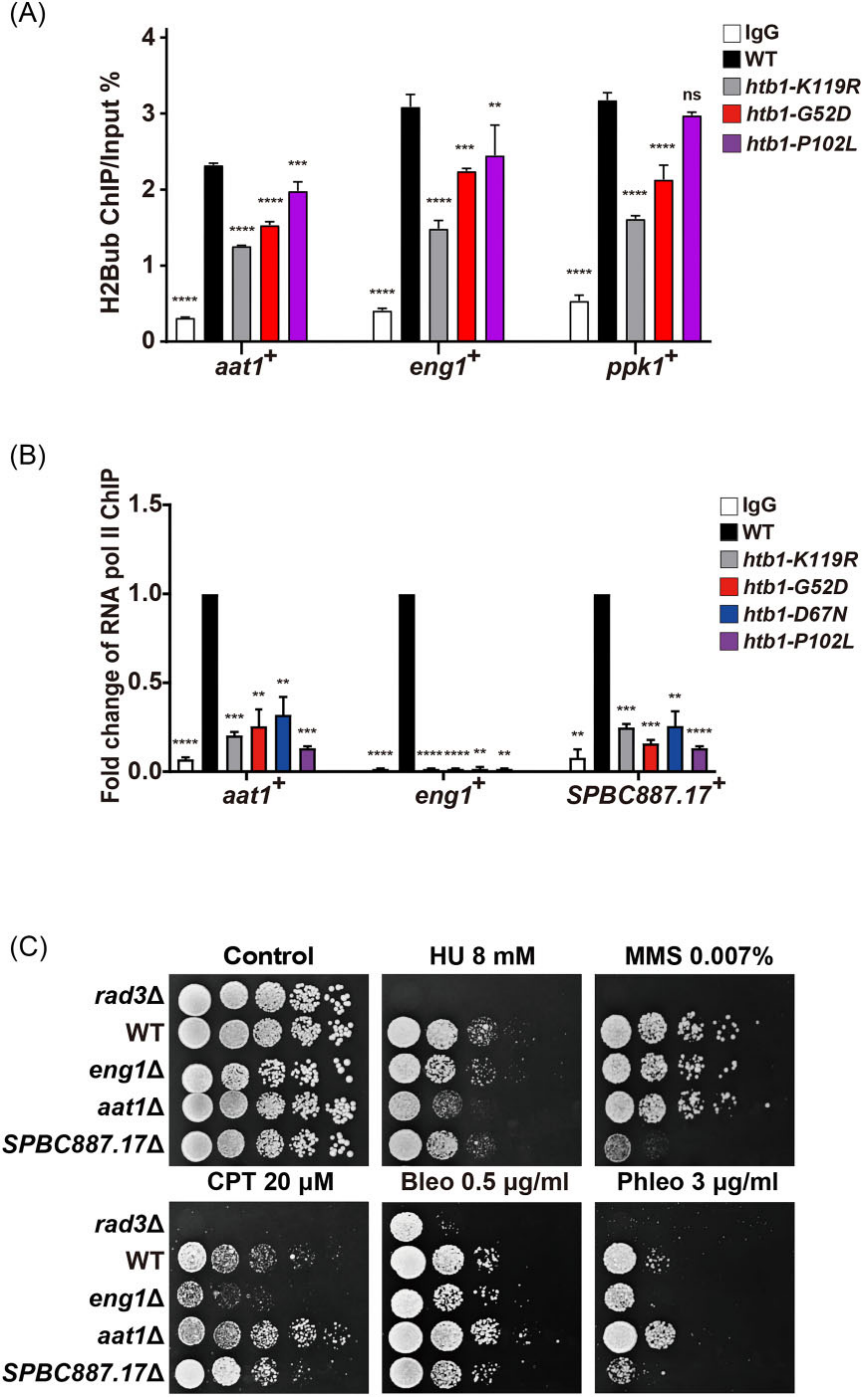
The ChIP-qPCR analysis of enrichment levels of H2B^ub^ and RNA pol II at the indicated genes in the *htb1-G52D*/*D67N*/*P102L* oncomutants, as well as the genotoxic phenotypes of those genes’ deletion mutants. (A) The enrichment percentage of H2B^ub^ at *aat1*^+^, *eng1*^+^, and *ppk1*^+^ genes is shown in *htb1-K119R* (YGF226), *htb1-G52D* (YGF277), and *htb1-P102L* (YGF279). (B) The RNA pol II subunit Rpb1 enrichment at *aat1*^+^, *eng1*^+^, and *SPBC887.17*^+^ genes in *htb1-K119R* (YGF226), *htb1-G52D* (YGF277), *htb1-D67N* (YGF324), and *htb1-P102L* (YGF279) is relative to that in WT (TK8) (set as 1). The H2B^ub^ enrichment or relative fold change of RNA pol II levels is plotted as mean ± SD (*n* = 3). A one-way ANOVA is performed for multiple comparisons to WT. (C) The genotoxic phenotypes of the *eng1*Δ (YGF651), *aat1*Δ (YGF652), and *SPBC887.17*Δ (YGF653) mutants. The length of incubation time is 4 days.

To test whether H2B^ub^ levels are diminished at these representative genes whose expression is decreased in the *htb1-G52D* and *htb1-P102L* mutants, we conducted an H2B^ub^-ChIP experiment, which was validated in our previous study (Qin et al. 2024). Consistently, H2B^ub^ levels were significantly enriched at the transcribed genes *act1*^+^ and *aro1*^+^ in WT compared with the *htb1-K119R* mutant, but not at the intergenic region *ars2004*, which is not transcribed and has few H2B^ub^ (Fig. S6A). H2B^ub^ levels were also reduced at *act1*^+^ and *aro1*^+^, but not at *ars2004*, in the *htb1-G52D* and *htb1-P102L* compared with WT (Fig. S6A). Importantly, we found that H2B^ub^ levels are decreased at *aat1*^+^, *eng1*^+^, and *ppk1*^+^ in the *htb1-G52D* and *htb1-P102L* mutants (Fig. 6A), supporting that H2B^ub^ regulates the expression of these genes.

Previous studies demonstrated that H2B^ub^ is associated with elongating RNA polymerase II (pol II) in *S. cerevisiae* and reduced gene expression in *htb1-K119R* mutant is correlated with a transcriptional elongation defect in *S. pombe* (Xiao et al. 2005, Tanny et al. 2007). To understand how H2B^ub^ regulates gene expression in *htb1-G52D*/*D67N/P102L*, we analyzed RNA pol II levels in the representative genes using a ChIP-qPCR assay. The results showed that RNA pol II levels at *aat1*^+^, *eng1*^+^, and *SPBC887.17*^+^ were significantly reduced in *htb1-K119R, htb1-G52D, htb1-D67N*, and *htb1-P102L* compared with WT (Fig. 6B). However, RNA pol II levels were unaltered at *act1*^+^ and *aro1*^+^ (Fig. S6B), whose expression is not regulated by H2B^ub^. Collectively, these data further indicate that the compromised H2B^ub^ downregulates gene expression in *htb1-G52D, htb1-D67N*, and *htb1-P102L* by perturbing RNA pol II elongation.

### The deletion of *aat1*^+^, *eng1*^+^, and *SPBC887.17*^+^ leads to genotoxic defects and their overexpression partially suppresses the temperature sensitivity of *htb1-G52D*

Previous large-scale studies have suggested that the *ppk1*Δ mutant is sensitive to HU and thiabendazole, which interferes with microtubule polymerization (Bimbó et al. 2005, Pan et al. 2012), and that *eng1*Δ is sensitive to MMS and bleomycin (Rodríguez-López et al. 2023). To substantiate this, we constructed *eng1*Δ, *aat1*Δ, and *SPBC887.17*Δ mutants and found that each was sensitive to at least one genotoxin (Fig. 6C). The *eng1*Δ exhibited sensitivities to CPT, bleomycin, and phleomycin. The *aat1*Δ showed the HU sensitivity. The *SPBC887.17*Δ was sensitive to MMS, bleomycin, and phleomycin (Fig. 6C). These data may partially explain why *htb1-G52D/D67N/P102L* with reduced levels of Aat1, Eng1, Ppk1, and SPBC887.17 exhibit genotoxic sensitivity.

To determine whether the reduced expression of a particular gene is sufficient for the genotoxic defect of *htb1-G52D* and *htb1-P102L*, we individually overexpressed *aat1*^+^, *eng1*^+^, *ppk1*^+^, and *SPBC887.17*^+^ from the moderate-strength *adh21* promoter in the *htb1-G52D* mutant. However, they failed to rescue the temperature and genotoxic sensitivities of *htb1-G52D* (Fig. S7A). To rule out the possibility that the relatively weak overexpression of Ppk1 was insufficient to compensate for the defective phenotypes of *htb1-G52D*, we additionally overexpressed *ppk1*^+^ from the strong-strength *adh11* promoter in WT, *htb1-G52D*, and *htb1-P102L* cells, but still observed no rescue effect (Fig. S7B). Furthermore, we overexpressed both *ppk1*^+^ and *aat1*^+^ from the *adh11* promoter and found that it partially rescued the temperature sensitivity of *htb1-G52D* but had no effect on genotoxic sensitivity (Fig. S7C). These data suggest that a set of altered transcripts controlled by H2B^ub^ may be required to contribute to the genomic instability of *htb1-G52D, htb1-D67N*, and *htb1-P102L*.

## Discussion

**Figure 7.**
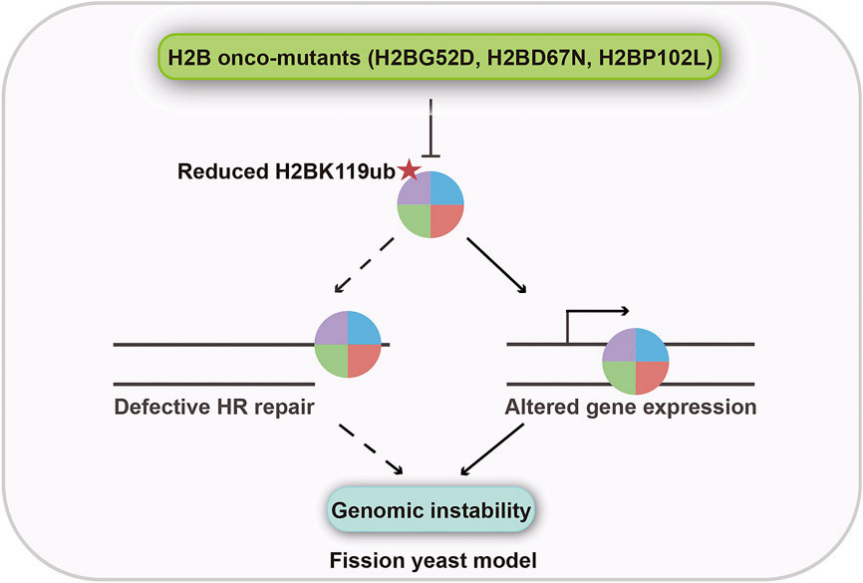
A unified model is proposed. The diverse oncomutants at H2B-Gly52/Asp67/Pro102 residues are modeled and characterized in the fission yeast *S. pombe*. Only the H2BG52D, H2BD67N, and H2BP102L exhibit significant genomic instability. Mechanistically, the compromised H2B^ub^ in H2BG52D/D67N/P102L plays a crucial role. It not only impairs the HR repair efficiency but also alters the genome-wide gene expression. The diagrams with dashed lines represent our previous findings (Qin et al. 2024), and those with solid lines represent the findings in this study.

We constructed diverse oncomutants of *htb1-Gly52/Asp67/Pro102* in *S. pombe* and characterized their genotoxin susceptibility phenotypes, H2B^ub^ levels, and gene expression. Among them, only *htb1-G52D, htb1-D67N*, and *htb1-P102L* exhibit significant sensitivities to high temperature and genotoxins. Correlatively, the levels of H2B^ub^ are notably reduced only in the *htb1-G52D, htb1-D67N*, and *htb1-P102L* mutants. Mechanistically, the transcriptomes in *htb1-G52D, htb1-D67N*, and *htb1-P102L* cells are altered, which are overlapping with H2B^ub^-regulated transcripts. These altered transcripts are largely restored to WT levels when H2B^ub^ levels are recovered in the absence of *ubp8*^+^. This coincides with the rescue of defective phenotypes in *htb1-G52D, htb1-D67N*, and *htb1-P102L* cells when H2B^ub^ levels are restored (Qin et al. 2024). Although we previously demonstrated that the compromised H2B^ub^ in *htb1-G52D* and *htb1-P102L* directly impairs Rad51 recruitment and causes HR repair defect, it does not generally affect the expression of DNA repair genes (Qin et al. 2024). Therefore, the alteration of genome-wide gene expression due to H2B^ub^ deficiency is a novel mechanism underlying the genomic instability of *htb1-G52D, htb1-D67N*, and *htb1-P102L* oncomutants. Collectively, we propose a model in which both HR repair and gene expression are two parallel and independent subpathways under the control of H2B^ub^, whose deficiency plays a determining role in the genomic instability caused by H2B^G52D^, H2B^D67N^, and H2B^P102L^ oncohistones (Fig. 7).

The *htb1-G52D/D67N/P102L* mutants are similar to the *htb1-G52E/D67Q/P102I* mutants in producing amino acid changes with comparable characteristics, but these have not been reported as oncohistones and exhibit mild or no genotoxic defects and almost normal H2B^ub^ levels. In another aspect, nononcohistone *htb1-D67A/P102A* mutants also behave like WT. Therefore, our mutation spectra analyses indicate that only specific amino acid changes among the *htb1-Gly52/Asp67/Pro102* diverse oncomutants, such as *htb1-G52D, htb1-D67N*, and *htb1-P102L*, have an impact on H2B^ub^ levels. The underlying reason is unclear. We previously indicated that the recruitment of Ubp8 onto the nucleosome is enhanced in the *htb1-G52D* mutant (Qin et al. 2024). Thus, we speculate that only the H2B^G52D/D67N/P102L^ changes can specifically remodel the nucleosome structure and promote the interaction of Ubp8 with the H2B in the nucleosome. To support this speculation, we predicted the nucleosome structures harboring diverse H2B-Gly52/Asp67/Pro102 changes. The negative charge of the H2B^G52D^ is supposed to weaken the H2B–DNA interaction with increased distance (Fig. S8A), which could destabilize the nucleosome and enhance nucleosome remodeling/sliding (Bagert et al. 2021, Jain and Strahl 2021). H2B^D67N^ is predicted to affect the contact with H4-Tyr98 on the histone dimer–tetramer interface (Fig. S8B), which may perturb histone exchange (Bagert et al. 2021, Jain and Strahl 2021). H2B^P102L^ could attenuate the hydrophobic interaction with H2A-Glu93/Lys96/Leu97 (Fig. S8C). However, the other H2B-Gly52/Asp67/Pro102 changes appear to have no such effects (Fig. S8A–C). The relevant structures need to be experimentally solved to test these predictions in the future.

This study revealed that the effects of different amino acid substitutions in oncohistone H2B on histone PTMs are generally associated with their oncogenic phenotypes such as genomic instability, which is consistent with the characteristic of oncohistone H3. Certain *htb1-Gly52* oncomutants do not affect H2B^ub^ levels, yet they still exhibit genotoxic sensitivity. However, this is not found in *htb1-Asp67/Pro102* oncomutants, where only the *htb1-D67N* and *htb1-P102L* affecting H2B^ub^ are defective in response to genotoxins. Our previous study on the synergistic genotoxic sensitivity of *htb1-G52D K119R* double mutant suggests that H2B^G52D^ not only reduces H2B^ub^ levels but also alters nucleosome remodeling independent of H2B^ub^. However, the lack of synergistic sensitivity between *htb1-D67N*/*P102L* and *htb1-K119R* suggests that H2B^D67N/P102L^ primarily affect H2B^ub^ levels (Qin et al. 2024). Therefore, we propose that certain *htb1-Gly52* mutants but not *htb1-Asp67/Pro102* mutants could display genotoxic sensitivity by affecting nucleosome remodeling (Mitchener and Muir 2022), which is independent of H2B^ub^ regulation. This phenomenon is consistent with studies on H3 oncohistones in which the alteration of H3 PTMs is also not the sole mechanism for their phenotypes (Caeiro et al. 2024).

With respect to the relationship between histone PTMs and gene expression, the reduction in H3K27 and H3K36 methylation levels could not be mainly responsible for the changed gene expression in H3K27M, H3G34R/W, and H3K36M oncomutants. However, the reduction in H2B^ub^ seems to be the main cause of altered gene expression in *htb1-G52D, htb1-D67N*, and *htb1-P102L*. Thus, the impacts of altered histone PTMs on gene expression may be different in diverse histone oncomutants, which encourages us to study more noncanonical oncohistones in the future.

The mechanism by which dysregulated gene expression contributes to the genomic instability in *htb1-G52D/D67N/P102L* is still unclear. The individual overexpression of *aat1*^+^, *ppk1^+^, eng1*^+^, and *SPBC887.17*^+^ fails to compensate for the temperature and genotoxic defects of *htb1-G52D*. Interestingly, the combined overexpression of *aat1*^+^ and *ppk1^+^* rescues the temperature sensitivity of *htb1-G52D*. Thus, the dysregulation of a particular transcript or protein could not be sufficient to contribute to genomic instability. Instead, we speculate that aberrant expression of a set of genes, which are regulated by H2B^ub^, may play a synergistic role in the genomic instability of *htb1-G52D/D67N/P102L*. Furthermore, a previous study suggested that altered expression of significantly clustered genes controlled by H2B^ub^ plays a specific role in the stress response (Tanny et al. 2007); thus, it is also possible that H2B^ub^-mediated alterations of gene expression in *htb1-G52D/D67N/P102L* impair the response to DNA replication and damage stress.

Two genetic approaches are used in yeast to model oncohistones in cancer (Zhang et al. 2023). A single allele of a histone gene can be introduced with oncomutations and the other alleles are kept intact or deleted, which express oncohistone mutants either in the presence or the absence of WT histone proteins. The yeast cells expressing the oncohistone within many WT histone proteins closely model cancer cells, where only one copy of many histone genes is mutated, and allow analysis of dominant-negative effects and phenotypes. Alternatively, the yeast cells expressing the oncohistone as the sole copy of the histone protein provide direct and clean characterization of oncohistone function as well as rapid and unbiased screening for recessive phenotypes (Zhang et al. 2023). In this study, oncomutations that occur in the sole H2B gene *htb1*^+^ in *S. pombe* cells amplify their effects in the absence of WT H2B protein, which provides an advantage for detecting even small impacts of H2B oncohistones. In our previous study, H2B^G52D/P102L^ in the presence of WT H2B protein also lead to genotoxic sensitivity and reduction in H2B^ub^  *in cis* (Qin et al. 2024), but the dominant-negative effect of reduced H2B^ub^ in local genes on their expression is still unclear and is under current investigation. In human cancers, only one of the many H2B gene copies is mutated. The effects of H2B^G52D/D67N/P102L^ on gene expression, if there is any effect, could be confined to a few genes with the incorporation of an H2B oncohistone. It is also unclear whether these limited alterations in gene expression contribute to genomic instability and oncogenesis. Thus, whether the findings in this study can be applied to human cancer cells needs to be investigated in the future.

## Conclusion

In summary, we extended our previous study on H2B oncohistones in *S. pombe* and revealed that the specific amino acid substitutions in *htb1-Gly52/Asp67/Pro102* oncomutants, such as *htb1-G52D, htb1-D67N*, and *htb1-P102L*, cause genotoxic sensitivity and genomic instability. The underlying mechanisms include defective HR repair and altered gene expression, both of which are caused by the reduction in H2B^ub^ levels. Therefore, H2B^ub^ levels reduced by H2B oncohistones play a key role in oncogenesis.

## Supplementary Material

foaf027_Supplemental_Files

## Data Availability

The RNA-seq data have been deposited to Gene Expression Omnibus (GEO) under accession numbers GSE267247 and GSE290173. The other original data and resources are available from the corresponding author upon request.
