# Supplementary material for: Characterization of oncohistone H2B variants in Schizosaccharomyces pombe reveals a key role of H2B monoubiquitination deficiency in genomic instability by altering gene expression
Source: FEMS Yeast Res. 2025 May 22;25:foaf027. doi: 10.1093/femsyr/foaf027 (PMC12128920; doi:10.1093/femsyr/foaf027)
Supplement: foaf027_Supplemental_Files [file foaf027_supplemental_files.zip › Supplement-revised.pdf]

Supplementary data

Characterization of oncohistone H2B variants in *Schizosaccharomyces pombe* reveals a key role of H2B monoubiquitination deficiency in genomic instability by altering gene expression

A list of the materials

Figures of S1-S8

Tables of S1-S4

Tables of S5-S10 are separate excel files

Supplementary figures

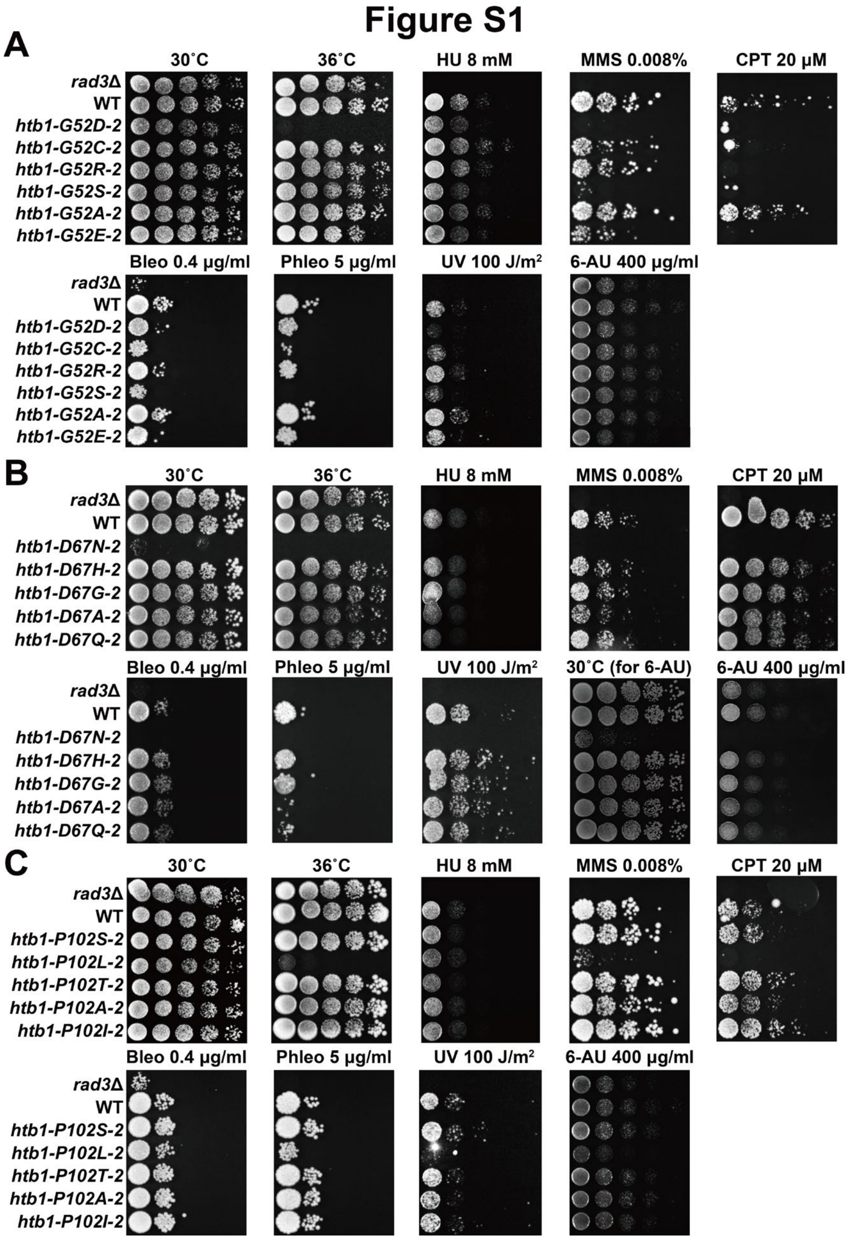

Figure S1 (related to Figure 1). The temperature and genotoxic phenotypes of the independent biological repeats of *htb1-Gly52/Asp67/Pro102* variants. (A) The growth of the second independent transformants of various *htb1-Gly52* mutants under indicated conditions. (B) The growth of the second independent transformants of various *htb1-Asp67* mutants under indicated conditions. (C) The growth of the second independent transformants of various *htb1-Pro102* mutants under indicated conditions. The *rad3*Δ (LD297) strain is a positive control. The length of incubation time is five days.

Figure S2

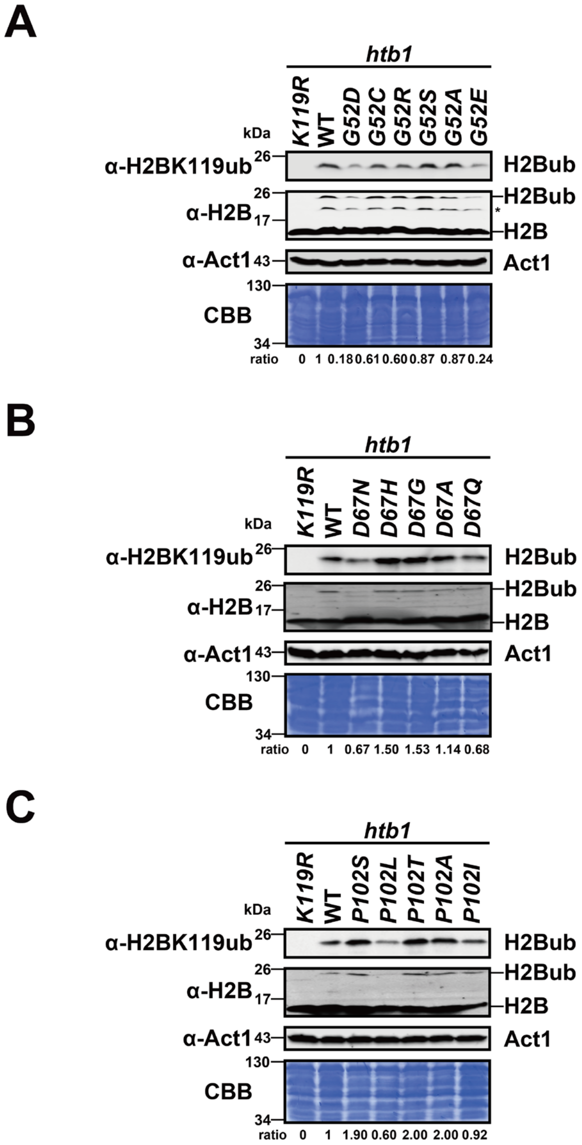

Figure S2 (related to Figure 2). The levels of H2Bub in independent biological repeats of *htb1-Gly52/Asp67/Pro102* diverse onco-mutants. (A) Immunoblots of H2Bub levels in second biological repeats of *htb1-K119R* (YGF226), WT (TK8), *htb1-G52D* (YGF277), *htb1-G52C* (YGF507), *htb1-G52R* (YGF510), *htb1-G52S* (YGF460), *htb1-G52A* (YGF459), and *htb1-G52E* (YGF508) cells. The Asterisk indicates non-specific bands. (B) Immunoblots of H2Bub levels in second biological repeats of *htb1-K119R* (YGF226), WT (TK8), *htb1-D67N* (YGF324), *htb1-D67H* (YGF515), *htb1-D67G* (YGF514), *htb1-D67A* (YGF513), and *htb1-D67Q* (YGF509) mutants. (C) Immunoblots of H2Bub levels in second biological repeats of *htb1-K119R* (YGF226), WT (TK8), *htb1-P102S* (YGF325), *htb1-P102L* (YGF279), *htb1-P102T* (YGF512), *htb1-P102A* (YGF511), and *htb1-P102I* (YGF505) cells. The intensity of the H2Bub band is normalized to that of CBB staining. The fold change of this normalization factor in the indicated mutants relative to WT (set as 1) is denoted as ratio at the bottom.

Figure S3

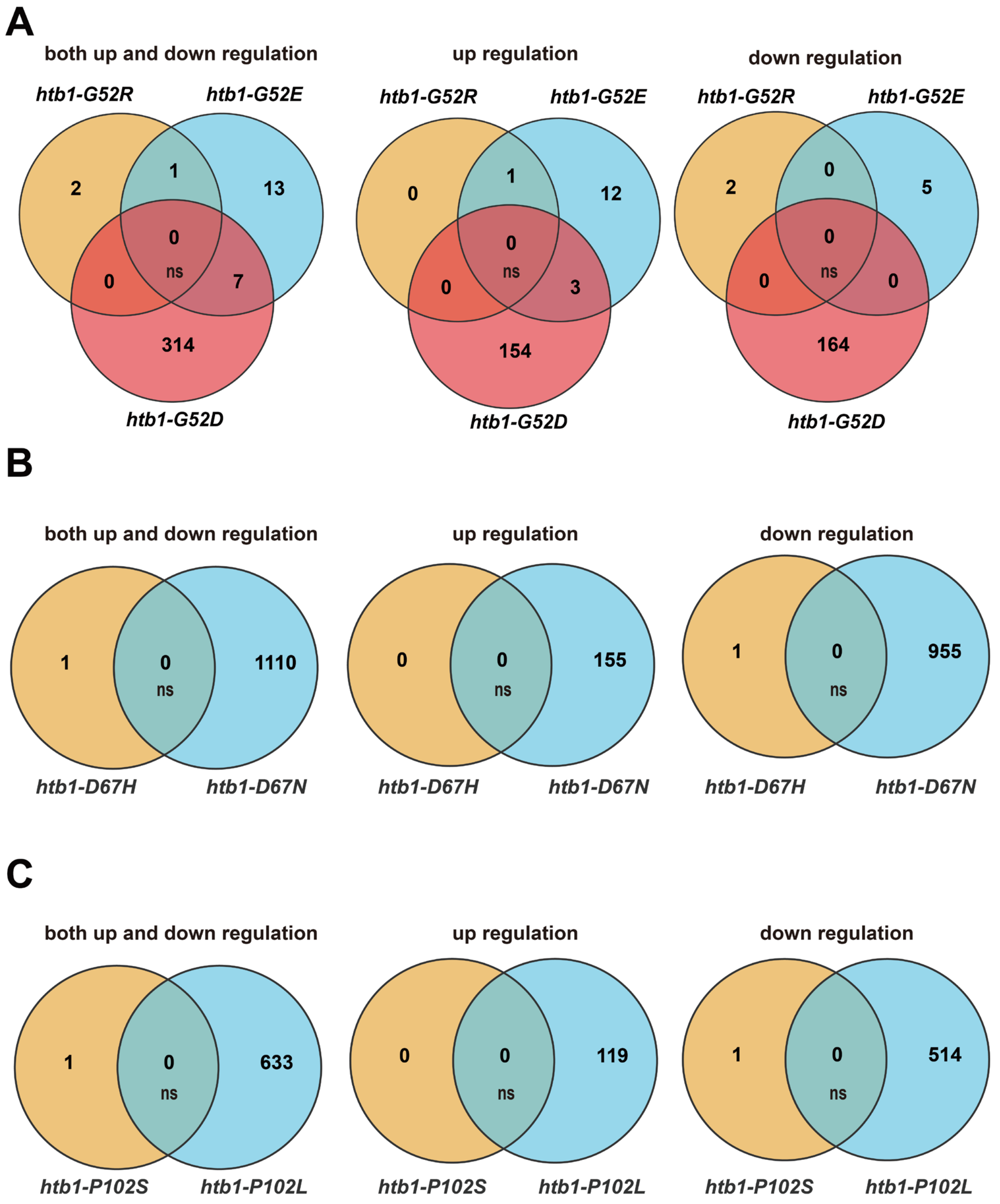
A
both up and down regulation
htb1-G52R
htb1-G52E
htb1-G52D
2
1
13
0
ns
0
7
314
up regulation
htb1-G52R
htb1-G52E
htb1-G52D
0
1
12
0
ns
0
3
154
down regulation
htb1-G52R
htb1-G52E
htb1-G52D
2
0
5
0
ns
0
0
164
B
both up and down regulation
1
0
ns
1110
htb1-D67H
htb1-D67N
up regulation
0
0
ns
155
htb1-D67H
htb1-D67N
down regulation
1
0
ns
955
htb1-D67H
htb1-D67N
C
both up and down regulation
1
0
ns
633
htb1-P102S
htb1-P102L
up regulation
0
0
ns
119
htb1-P102S
htb1-P102L
down regulation
1
0
ns
514
htb1-P102S
htb1-P102L

Figure S3

D

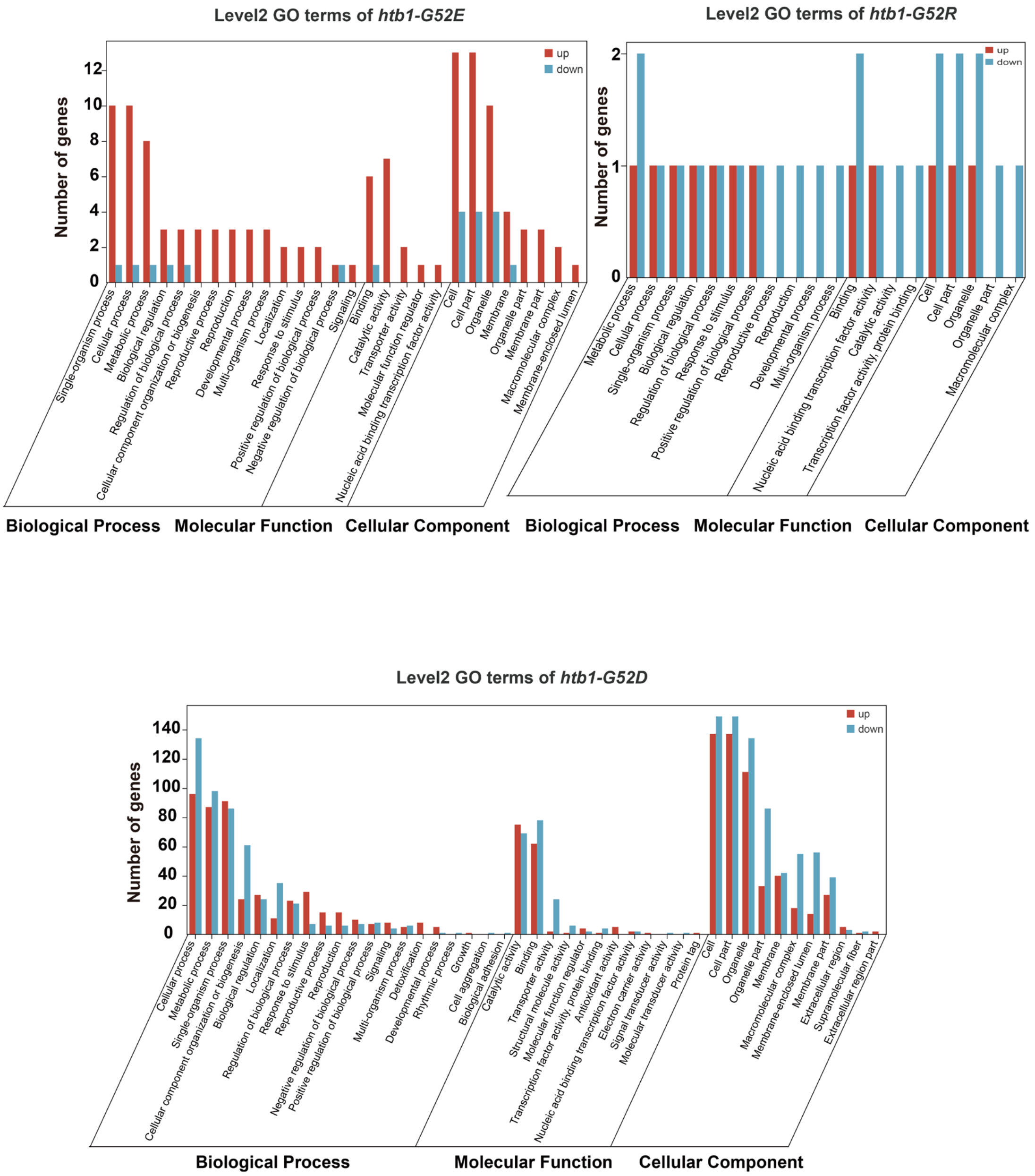

Level2 GO terms of htb1-G52E
Number of genes
up
down
Single-organism process
Cellular process
Metabolic process
Biological regulation
Regulation of biological process
Cellular component organization or biogenesis
Reproductive process
Reproduction
Developmental process
Multi-organism process
Localization
Response to stimulus
Positive regulation of biological process
Negative regulation of biological process
Signaling
Binding
Catalytic activity
Transporter activity
Molecular function regulator
Nucleic acid binding transcription factor activity
Cell
Cell part
Organelle
Membrane
Organelle part
Membrane part
Macromolecular complex
Membrane-enclosed lumen
Biological Process
Molecular Function
Cellular Component
Level2 GO terms of htb1-G52R
Number of genes
up
down
Metabolic process
Cellular process
Single-organism process
Biological regulation
Regulation of biological process
Response to stimulus
Positive regulation of biological process
Reproductive process
Reproduction
Developmental process
Multi-organism process
Binding
Nucleic acid binding transcription factor activity
Catalytic activity
Transcription factor activity, protein binding
Cell
Cell part
Organelle
Organelle part
Macromolecular complex
Biological Process
Molecular Function
Cellular Component
Level2 GO terms of htb1-G52D
Number of genes
up
down
Cellular process
Metabolic process
Single-organism process
Cellular component organization or biogenesis
Biological regulation
Localization
Regulation of biological process
Response to stimulus
Reproductive process
Reproduction
Negative regulation of biological process
Positive regulation of biological process
Signaling
Multi-organism process
Detoxification
Developmental process
Rhythmic process
Growth
Cell aggregation
Biological adhesion
Catalytic activity
Binding
Transporter activity
Structural molecule activity
Molecular function regulator
Transcription factor activity, protein binding
Antioxidant activity
Nucleic acid binding transcription factor activity
Electron carrier activity
Signal transducer activity
Molecular transducer activity
Protein tag
Cell
Cell part
Organelle
Organelle part
Membrane
Macromolecular complex
Membrane-enclosed lumen
Membrane part
Extracellular region
Supramolecular fiber
Extracellular region part
Biological Process
Molecular Function
Cellular Component

Figure S3

E

Level2 GO terms of *htb1-D67H*

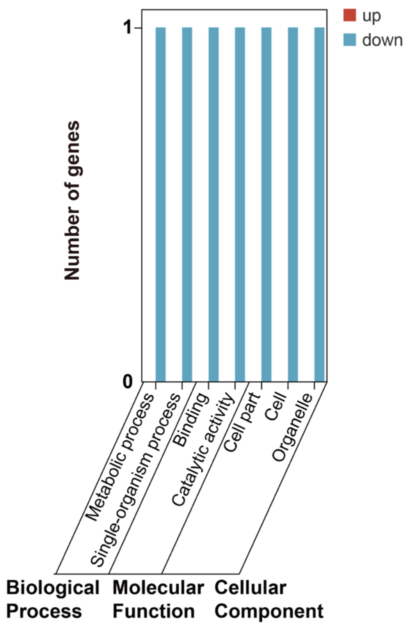

Level2 GO terms of *htb1-D67N*

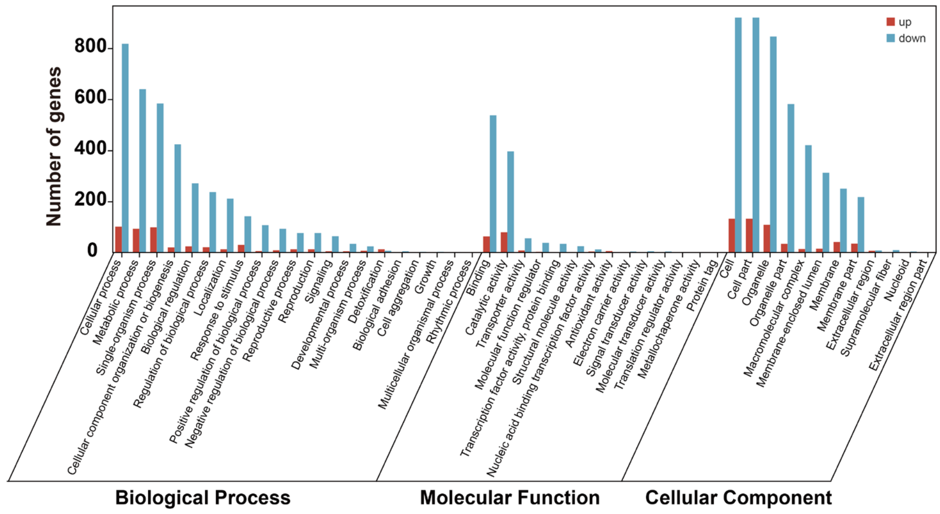

Figure S3

F

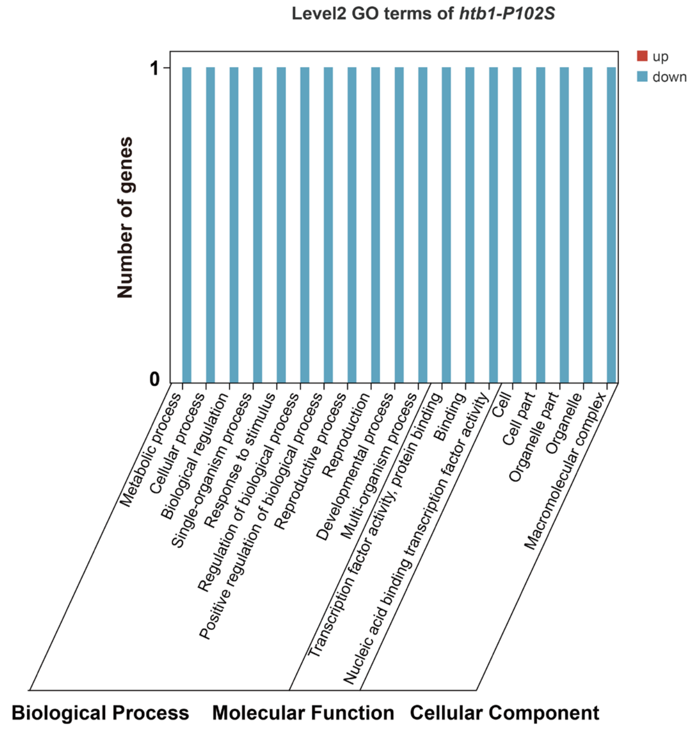

Level2 GO terms of htb1-P102S
Number of genes
1
0
up
down
Metabolic process
Cellular process
Biological regulation
Single-organism process
Response to stimulus
Regulation of biological process
Positive regulation of biological process
Reproductive process
Reproduction
Developmental process
Multi-organism process
Transcription factor activity, protein binding
Binding
Nucleic acid binding transcription factor activity
Cell
Cell part
Organelle part
Organelle
Macromolecular complex
Biological Process
Molecular Function
Cellular Component

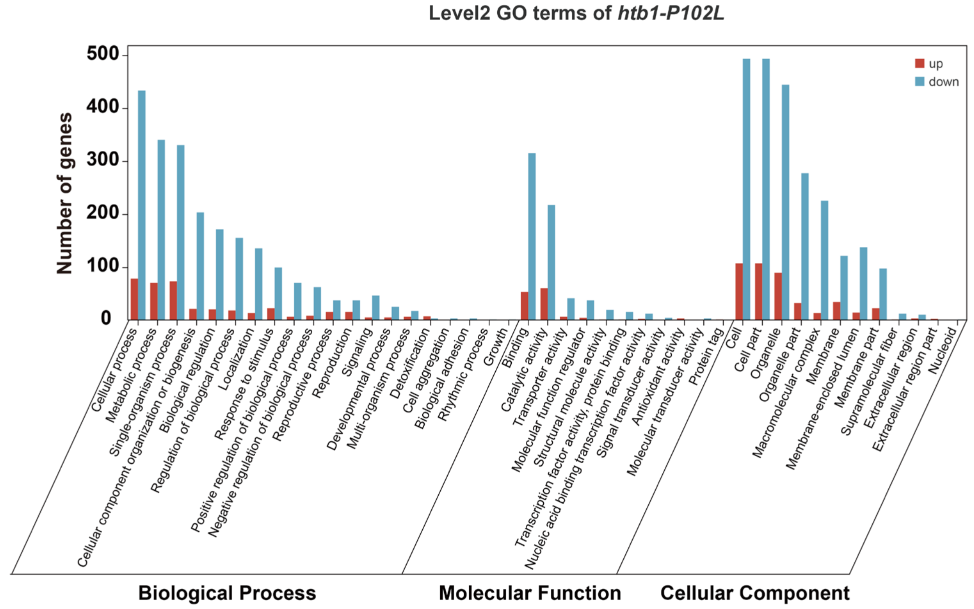

Level2 GO terms of htb1-P102L
Number of genes
500
400
300
200
100
0
up
down
Cellular process
Metabolic process
Single-organism process
Cellular component organization or biogenesis
Biological regulation
Regulation of biological process
Localization
Response to stimulus
Positive regulation of biological process
Negative regulation of biological process
Reproductive process
Reproduction
Signaling
Developmental process
Multi-organism process
Detoxification
Cell aggregation
Biological adhesion
Rhythmic process
Growth
Binding
Catalytic activity
Transporter activity
Molecular function regulator
Structural molecule activity
Transcription factor activity, protein binding
Nucleic acid binding transcription factor activity
Signal transducer activity
Antioxidant activity
Molecular transducer activity
Protein tag
Cell
Cell part
Organelle
Organelle part
Macromolecular complex
Membrane
Membrane-enclosed lumen
Membrane part
Supramolecular fiber
Extracellular region
Extracellular region part
Nucleoid
Biological Process
Molecular Function
Cellular Component

Figure S3

G

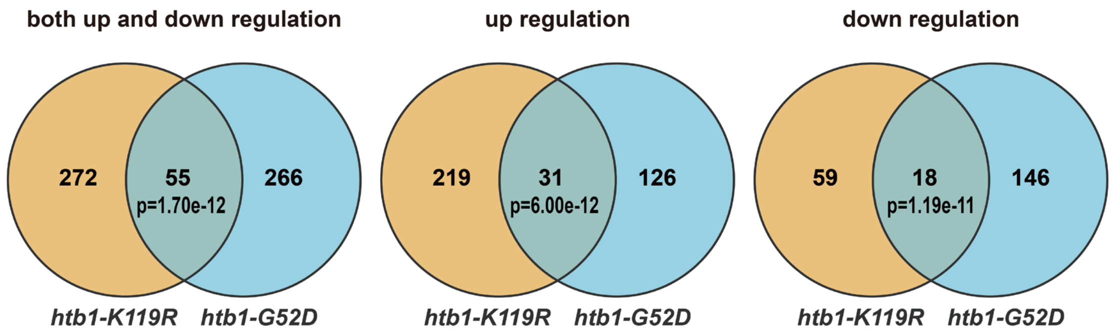

H

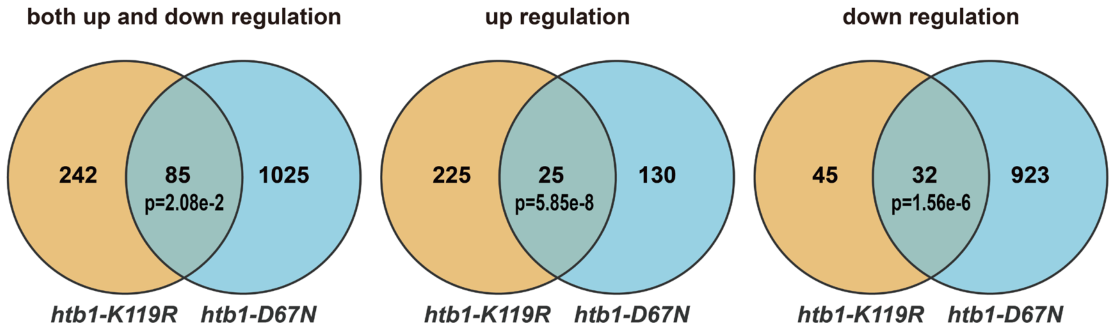

I

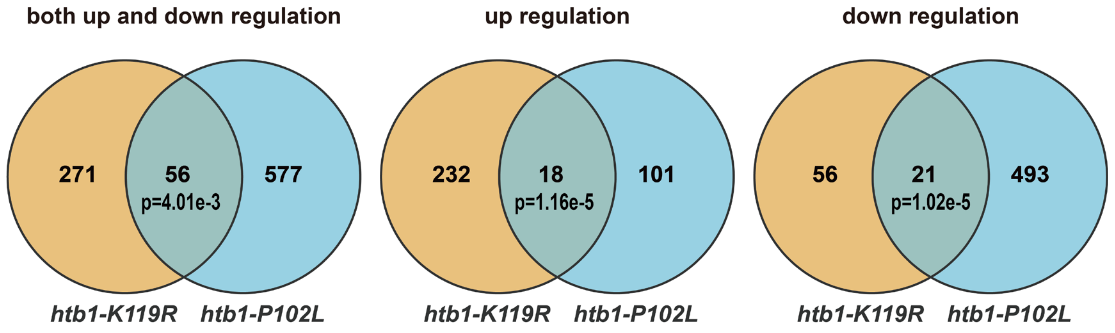

Figure S3

J

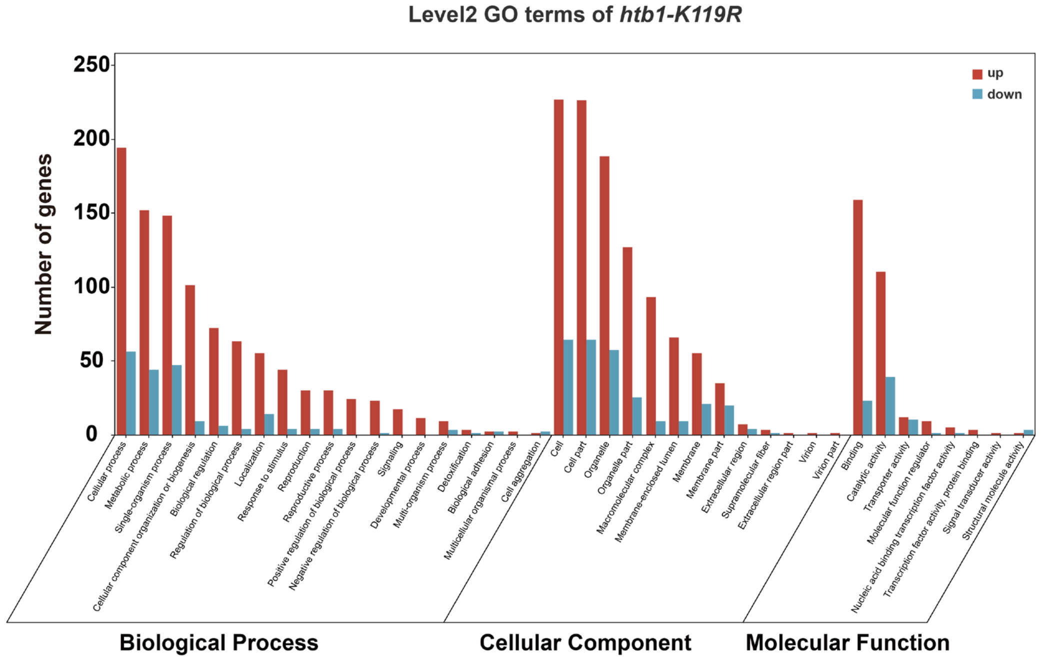

Figure S3 (related to Figure 3). Venn Diagrams and Level2 GO terms of DEGs in the representative *htb1-Gly52/Asp67/Pro102* onco-mutants and *htb1-K119R*. (A) Venn Diagrams of DEGs in *htb1-G52D* (YGF277), *htb1-G52R* (YGF510), and *htb1-G52E* (YGF508). (B) Venn Diagrams of DEGs in *htb1-D67N* (YGF324) and *htb1-D67H* (YGF515). (C) Venn Diagrams of DEGs in *htb1-P102S* (YGF325) and *htb1-P102L* (YGF279). (D) Cluster analysis of the GO level2 terms of biological process, molecular function, and cellular component in *htb1-G52D* (YGF277), *htb1-G52R* (YGF510), and *htb1-G52E* (YGF508) mutants. (E) Cluster analysis of the GO level2 terms of biological process, molecular function, and cellular component in *htb1-D67N* (YGF324) and *htb1-D67H* (YGF515) mutants. (F) Cluster analysis of the GO level2 terms in biological process, molecular function, and cellular component in *htb1-P102S* (YGF325) and *htb1-P102L* (YGF279) mutants. (G) Venn Diagrams of DEGs in *htb1-G52D* (YGF277) and *htb1-K119R* (YGF226). (H) Venn Diagrams of DEGs in *htb1-D67N* (YGF324) and *htb1-K119R* (YGF226). (I) Venn Diagrams of DEGs in *htb1-P102L* (YGF279) and *htb1-K119R* (YGF226). (J) Cluster analysis of the GO level2 terms in biological process, molecular function, and cellular component in *htb1-K119R* (YGF226) mutant. The Fisher's exact test is used for calculating p values of Venn Diagrams.

Figure S4

A

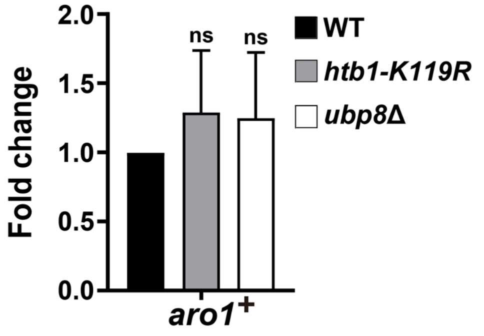

B

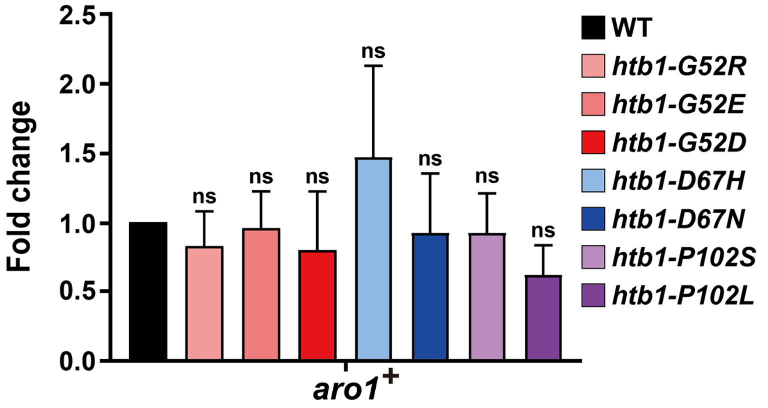

Figure S4 (related to Figure 4). (A) RT-qPCR analysis of *aro1*$^{+}$ transcript in *htb1-K119R* (YGF226) and *ubp8*Δ (YGF415) mutants. (B) RT-qPCR analysis of *aro1*$^{+}$ transcript in the indicated *htb1-Gly52/Asp67/Pro102* onco-mutants. The fold change of gene expression in the indicated mutants relative to that of WT (set as 1) is shown as mean ± SD (n = 3). A one-way ANOVA is performed to compare multiple data sets to WT.

Figure S5

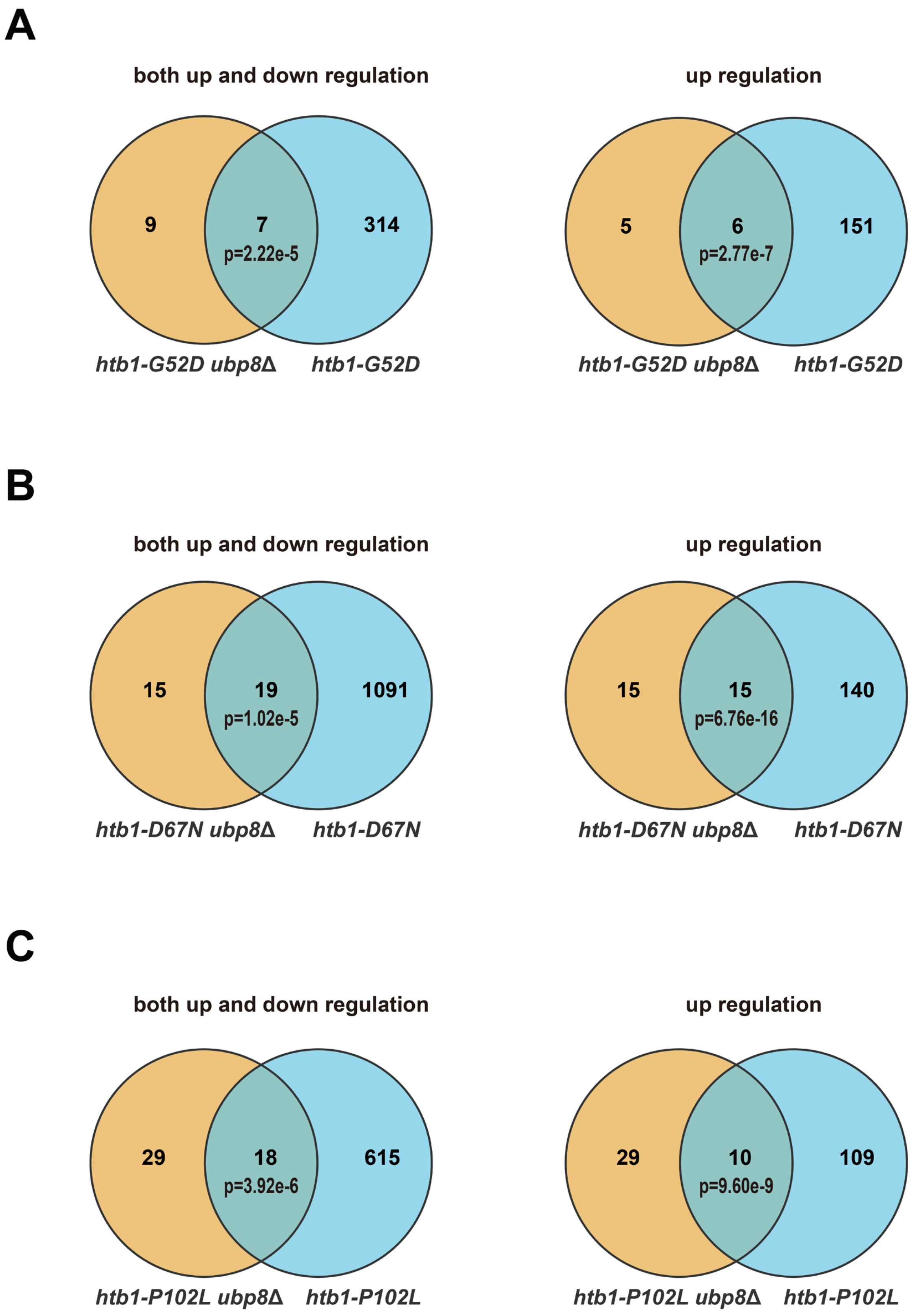
A
both up and down regulation
9
7
p=2.22e-5
314
htb1-G52D ubp8Δ
htb1-G52D
up regulation
5
6
p=2.77e-7
151
htb1-G52D ubp8Δ
htb1-G52D
B
both up and down regulation
15
19
p=1.02e-5
1091
htb1-D67N ubp8Δ
htb1-D67N
up regulation
15
15
p=6.76e-16
140
htb1-D67N ubp8Δ
htb1-D67N
C
both up and down regulation
29
18
p=3.92e-6
615
htb1-P102L ubp8Δ
htb1-P102L
up regulation
29
10
p=9.60e-9
109
htb1-P102L ubp8Δ
htb1-P102L

Figure S5

D

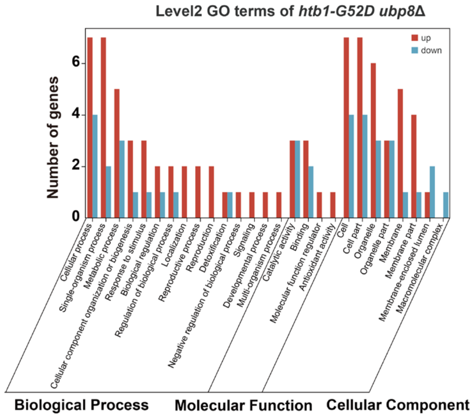

E

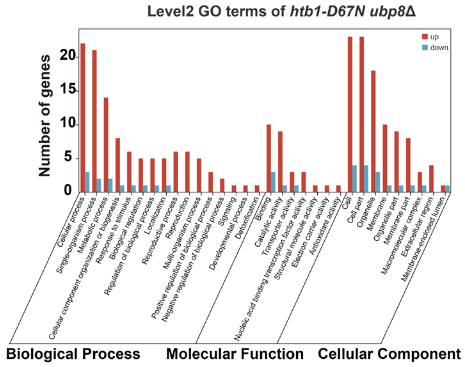

F

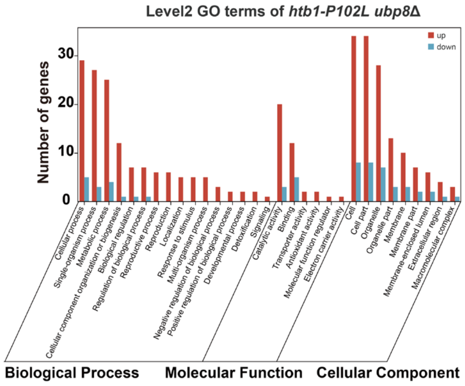

Figure S5 (related to Figure 5). Level2 GO terms of DEGs in the double mutants of *htb1-G52D*/*D67N*/*P102L* and *ubp8*Δ. (A) Venn Diagrams of the indicated DEGs in *htb1-G52D* (YGF277) and *htb1-G52D ubp8*Δ (YGF416). (B) Venn Diagrams of the indicated DEGs in *htb1-D67N* (YGF324) and *htb1-D67N ubp8*Δ (YGF443). (C) Venn Diagrams of the indicated DEGs in *htb1-P102L* (YGF279) and *htb1-P102L ubp8*Δ (YGF417). The Fisher's exact test is used for calculating *p* values of Venn Diagrams. (D) Cluster analysis of the GO level2 terms of biological process, molecular function, and cellular component in the *htb1-G52D ubp8*Δ (YGF416) mutant. (E) Cluster analysis of the GO level2 terms of biological process, molecular function, and cellular component in *htb1-D67N ubp8*Δ (YGF443). (F) Cluster analysis of the GO level2 terms of biological process, molecular function, and cellular component in *htb1-P102L ubp8*Δ (YGF417) mutants.

Figure S6

A

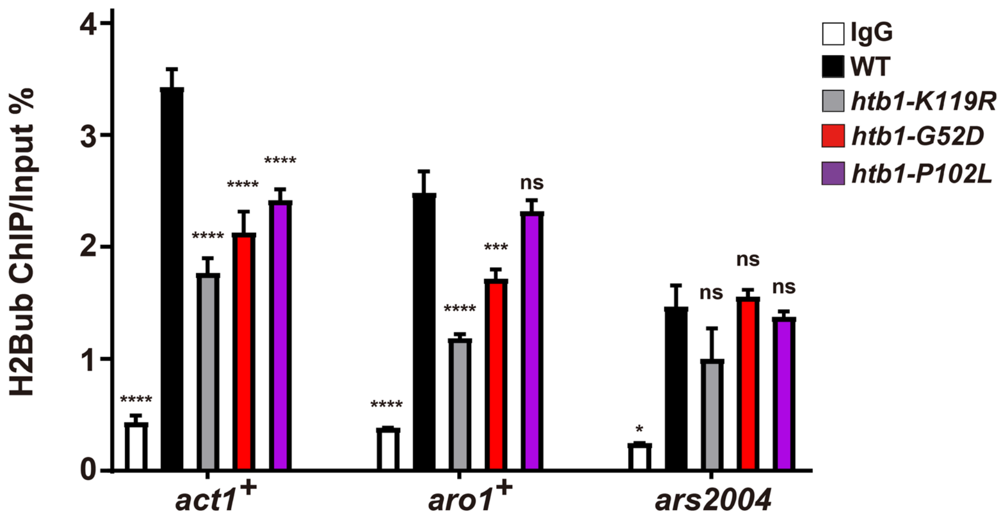

B

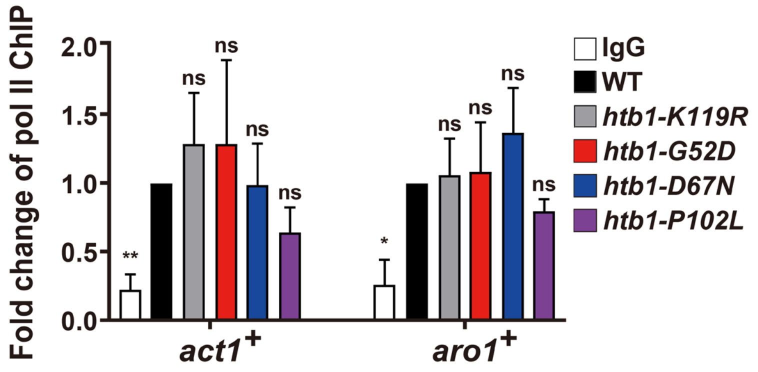

Figure S6 (related to Figure 6). (A) The ChIP-qPCR analysis of $H2B^{ub}$ enrichment percentage at *act1*$^+$, *aro1*$^+$, and an intergenic site *ars2004* in the *htb1-G52D/P102L/K119R* mutants. (B) The ChIP-qPCR analysis of relative fold change of RNA pol II subunit Rpb1 at *act1*$^+$ and *aro1*$^+$ genes in the *htb1-G52D/D67N/P102L/K119R* mutants. The RNA pol II enrichment in *htb1-K119R* (YGF226), *htb1-G52D* (YGF277), *htb1-D67N* (YGF324), and *htb1-P102L* (YGF279) is relative to that in WT (TK8) (set as 1). The H2Bub enrichment or relative fold change of RNA pol II is plotted as mean ± SD (n = 3). A one-way ANOVA is performed for multiple comparisons to WT.

Figure S7

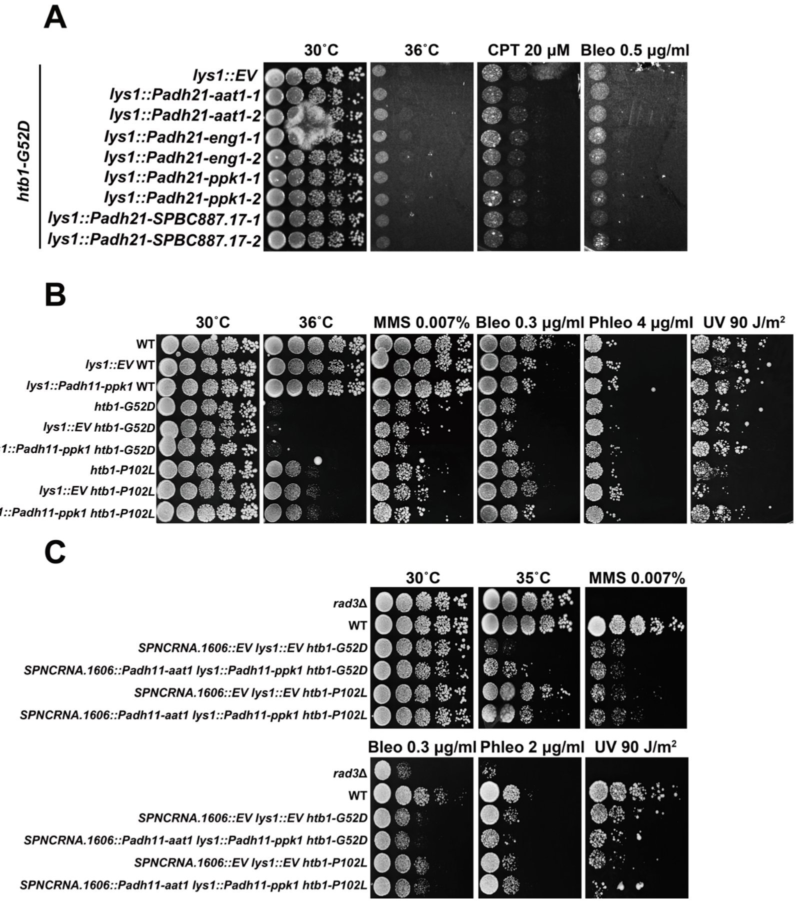

Figure S7. The effects of *aat1*$^+$, *eng1*$^+$, *ppk1*$^+$, and *SPBC887.17*$^+$ overexpression on the temperature and genotoxic sensitivities of the *htb1-G52D/P102L* mutants. (A) The growth of *htb1-G52D* with EV control (YGF422), *htb1-G52D* with overexpressed Aat1 (YGF530), *htb1-G52D* with overexpressed Eng1 (YGF531), *htb1-G52D* with overexpressed Ppk1 (YGF532), and *htb1-G52D* with overexpressed SPBC887.17 (YGF533) under indicated conditions. These genes are integrated at the *lys1* locus and overexpressed under *adh21* promoter. EV indicates empty vector. (B) The growth of WT (TK8), WT with overexpressed EV control (YGF467), WT with overexpressed Ppk1 (YGF470), *htb1-G52D* (YGF277), *htb1-G52D* with EV control (YGF422), *htb1-G52D* with overexpressed Ppk1 (YGF599), *htb1-P102L* (YGF279), *htb1-P102L* with EV control (YGF424), and *htb1-P102L* with overexpressed Ppk1 (YGF602) under indicated conditions. The *ppk1*$^+$ gene is integrated at the *lys1* locus and overexpressed under *adh11* promoter. (C) The growth of WT (TK8), *htb1-G52D* with EV control (YGF655), *htb1-G52D* with overexpressed Ppk1 and Aat1 (YGF657), *htb1-P102L* with EV control (YGF656), and *htb1-P102L* with overexpressed Ppk1 and Aat1 (YGF658) under indicated conditions. The *ppk1*$^+$ gene is integrated at the *lys1* locus and overexpressed under *adh11* promoter. The *aat1*$^+$ gene is integrated at the *SPNCRNA.1606* locus and overexpressed under *adh11* promoter. The length of incubation time is four days.

Figure S8

A

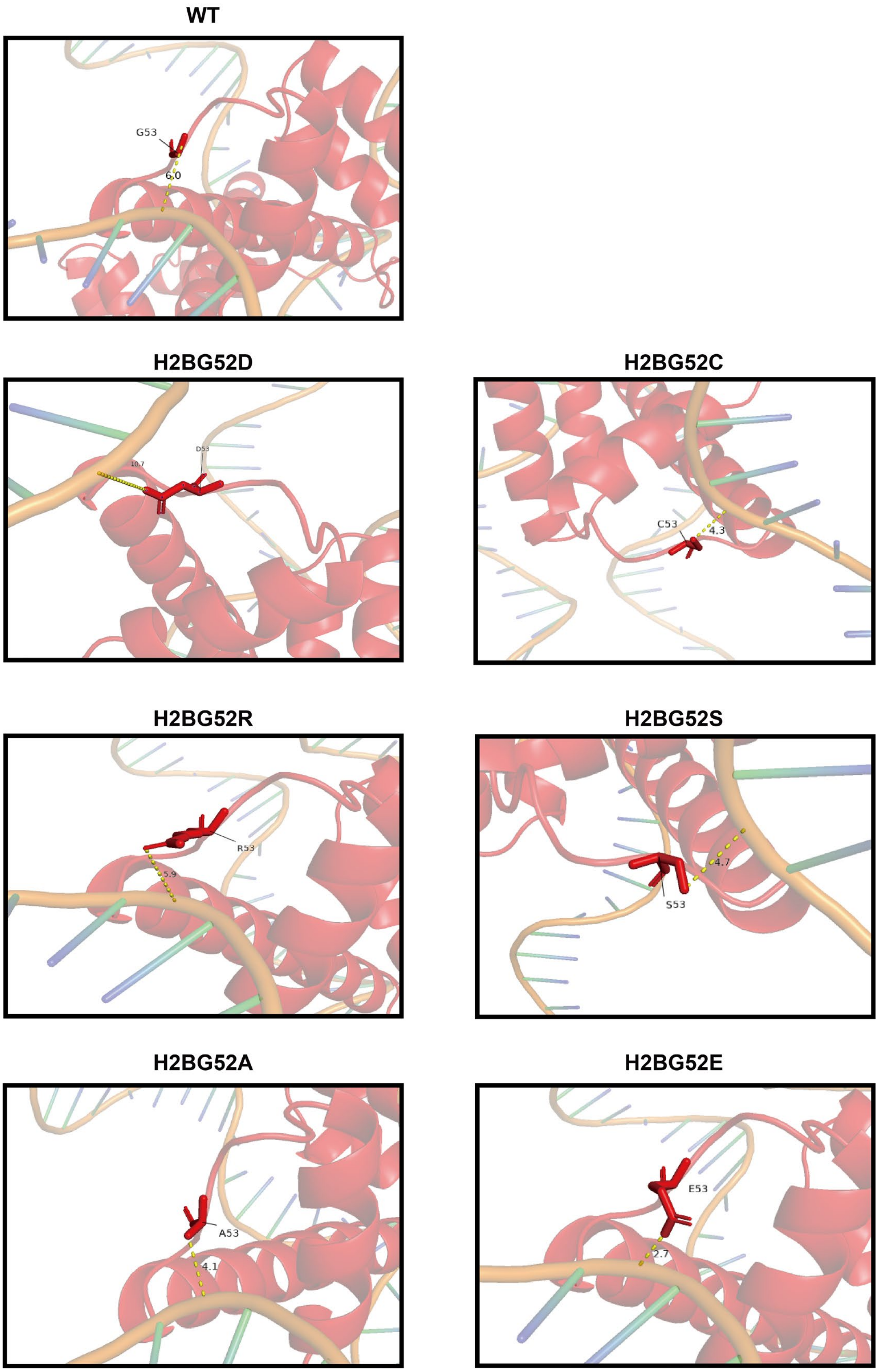

WT
G53
6.0
H2BG52D
D53
10.7
H2BG52C
C53
4.3
H2BG52R
R53
5.9
H2BG52S
4.7
S53
H2BG52A
A53
4.1
H2BG52E
E53
2.7

Figure S8

B

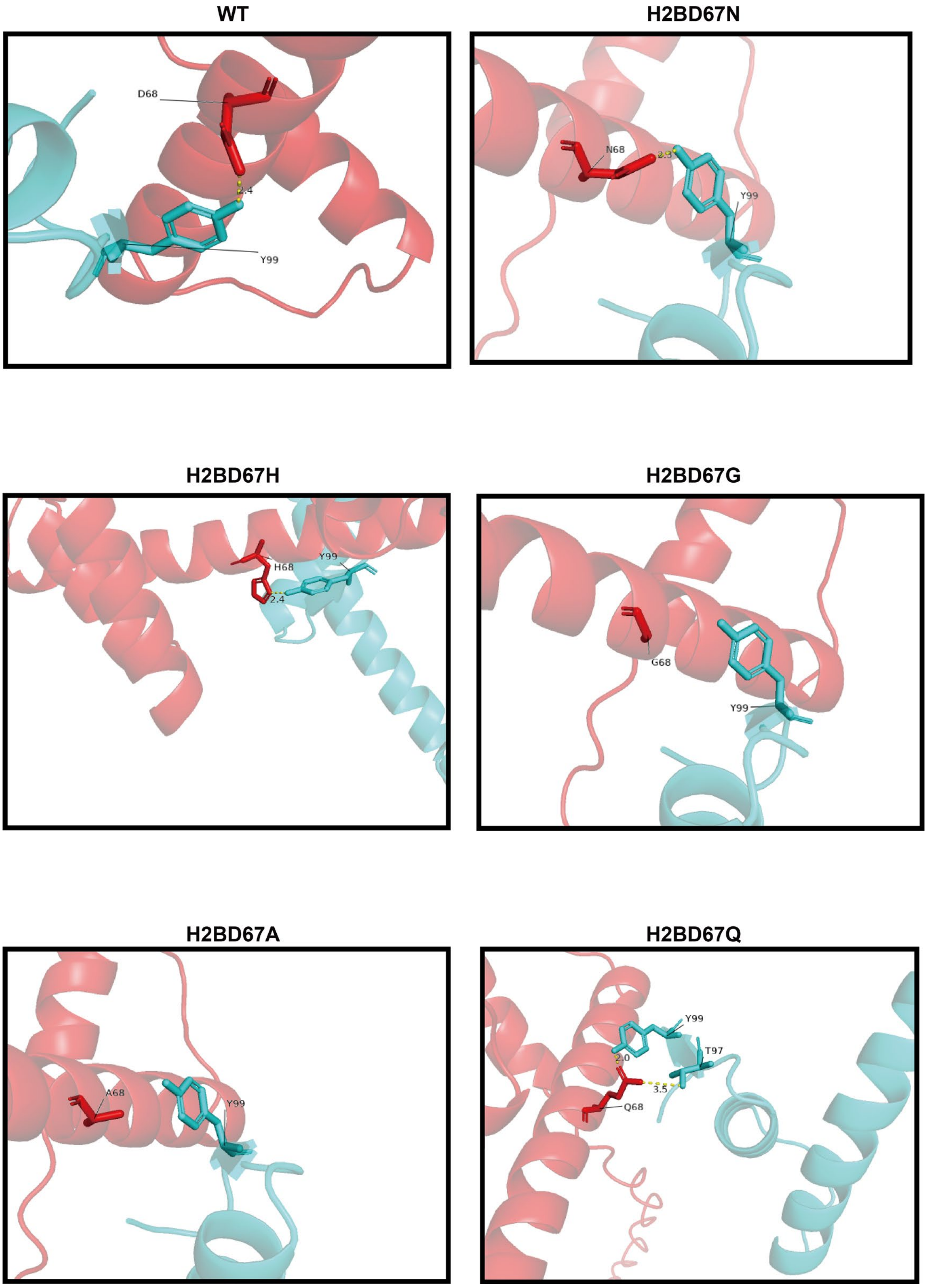
WT
D68
2.4
Y99
H2BD67N
N68
2.3
Y99
H2BD67H
H68
Y99
2.4
H2BD67G
G68
Y99
H2BD67A
A68
Y99
H2BD67Q
Y99
T97
2.0
3.5
Q68

Figure S8

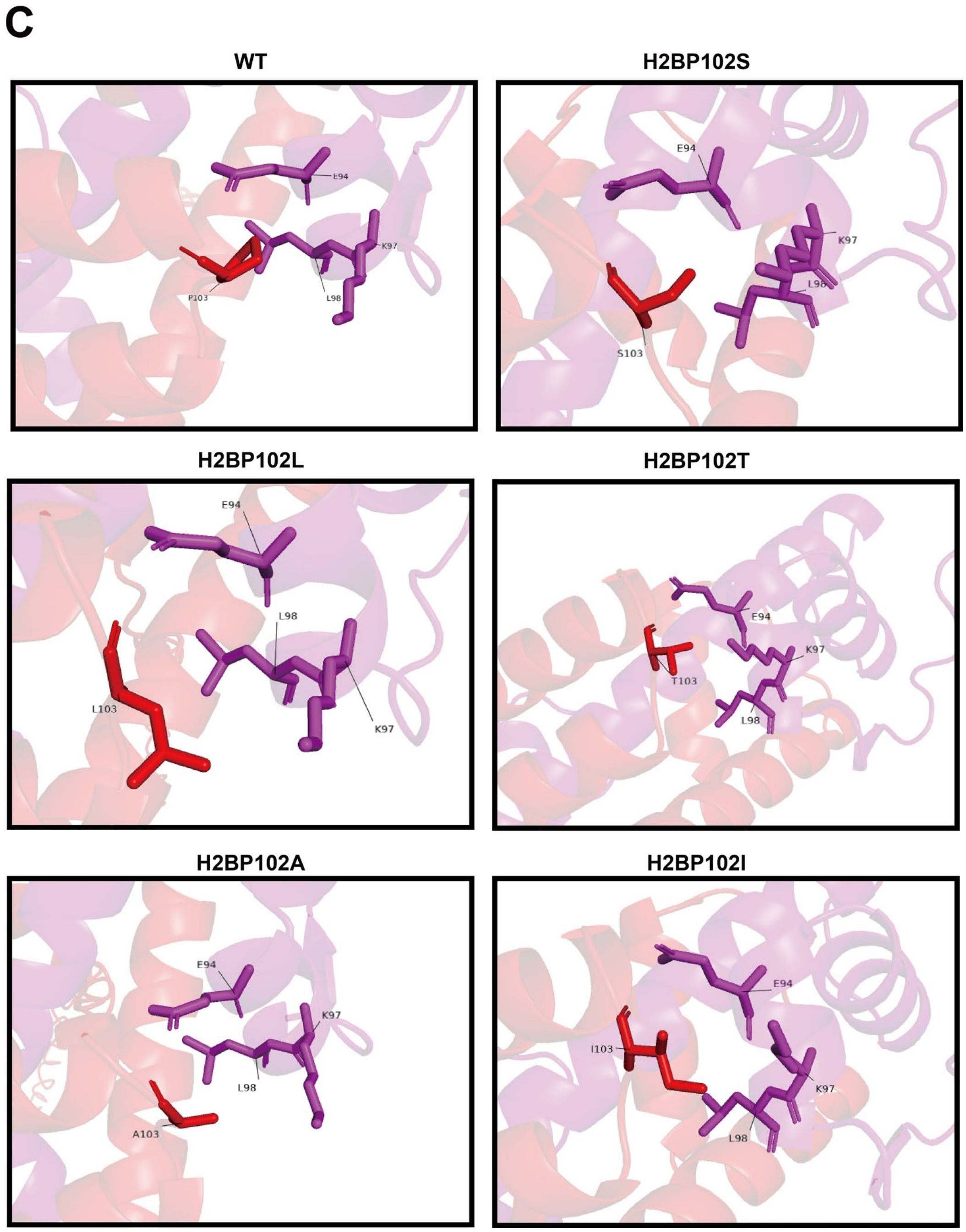

Figure S8. The predicated effects of onco-mutations at H2B-Gly52/Asp67/Pro102 residues on the *S. pombe* nucleosome structure. *S. pombe* histones H3 (P09988), H4 (P09322), H2A (P04909), and H2B (P04913) were used to predict the nucleosome structure by AlphaFold 3 server. (A) The predicted molecular docking between the mutated amino acids at H2B-Gly52 (red) and nucleosomal DNA (orange). (B) The predicted molecular docking between the mutated amino acids at H2B-Asp67 (red) and H4-Tyr98 (green). (C) The predicted molecular docking between the mutated amino acids at H2B-Pro102 (red) and H2A-Glu93/Lys96/Leu97 (purple).

Table S1. Yeast strains used in this study.

Strain	Genotype	Source
LD297	h^- *leu1-32 ura4-D18 ade6-M210 his3-D1 rad3Δ::LEU2*	L. Du
TK8	h^+ *ade6-M216 leu1-32 ura4-D18*	T. Kelly
YGF226	h^- *ura4-D18 htb1-K119R::kanMX6*	This study
YGF277	h^- *ade6? leu1-32 ura4-D18 htb1-G52D*	This study
YGF279	h^- *ade6? leu1-32 ura4-D18 htb1-P102L*	This study
YGF324	h^+ *ade6-M210 leu1-32 ura4-D18 htb1-D67N::kanMX6*	This study
YGF325	h^+ *ade6-M210 leu1-32 ura4-D18 htb1-P102S::kanMX6*	This study
YGF415	h^+ *ade6-M216 leu1-32 ura4-D18 ubp8Δ::kanMX6*	This study
YGF416	h^- *ade6? leu1-32 ura4-D18 htb1-G52D ubp8Δ::kanMX6*	This study
YGF417	h^- *ade6? leu1-32 ura4-D18 htb1-P102L ubp8Δ::kanMX6*	This study
YGF422	h^+ *ade6? leu1-32 ura4-D18 htb1-G52D lys1Δ::[pHBKA21-5FLAG-hphMX6]*	This study
YGF424	h^+ *ade6? leu1-32 ura4-D18 htb1-P102L lys1Δ::[pHBKA21-5FLAG-hphMX6]*	This study
YGF443	*h? ade6? leu1? ura4? htb1-D67N::kanMX6 ubp8Δ::hphMX6*	This study
YGF459	h^+ *ade6-M216 leu1-32 ura4-D18 htb1-G52A::kanMX6*	This study
YGF460	h^+ *ade6-M216 leu1-32 ura4-D18 htb1-G52S::kanMX6*	This study
YGF467	h^+ *ade6-M216 leu1-32 ura4-D18 lys1Δ::[pHBKA21-5FLAG-hphMX6]*	This study
YGF470	h^+ *ade6-M216 leu1-32 ura4-D18 lys1Δ::[pHBKA11-ppk1-5FLAG-hphMX6]*	This study
YGF505	h^+ *ade6-M216 leu1-32 ura4-D18 htb1-P102I::kanMX6*	This study
YGF506	h^+ *ade6-M216 leu1-32 ura4-D18 htb1-G52P::kanMX6*	This study
YGF507	h^+ *ade6-M216 leu1-32 ura4-D18 htb1-G52C::kanMX6*	This study
YGF508	h^+ *ade6-M216 leu1-32 ura4-D18 htb1-G52E::kanMX6*	This study
YGF509	h^+ *ade6-M216 leu1-32 ura4-D18 htb1-D67Q::kanMX6*	This study
YGF510	h^+ *ade6-M216 leu1-32 ura4-D18 htb1-G52R::kanMX6*	This study
YGF511	h^+ *ade6-M216 leu1-32 ura4-D18 htb1-P102A::kanMX6*	This study
YGF512	h^+ *ade6-M216 leu1-32 ura4-D18 htb1-P102T::kanMX6*	This study
YGF513	h^+ *ade6-M216 leu1-32 ura4-D18 htb1-D67A::kanMX6*	This study
YGF514	h^+ *ade6-M216 leu1-32 ura4-D18 htb1-D67G::kanMX6*	This study
YGF515	h^+ *ade6-M216 leu1-32 ura4-D18 htb1-D67H::kanMX6*	This study
YGF530	h^+ *ade6? leu1-32 ura4-D18 htb1-G52D lys1Δ::[pHBKA21-aat1-5FLAG-hphMX6]*	This study
YGF531	h^+ *ade6? leu1-32 ura4-D18 htb1-G52D lys1Δ::[pHBKA21-eng1-5FLAG-hphMX6]*	This study
YGF532	h^+ *ade6? leu1-32 ura4-D18 htb1-G52D lys1Δ::[pHBKA21-ppk1-5FLAG-hphMX6]*	This study
YGF533	h^+ *ade6? leu1-32 ura4-D18 htb1-G52D lys1Δ::[pHBKA21-SPBC887.17-5FLAG-hphMX6]*	This study

YGF591	h^+ *ade6-M216 leu1-32 ura4-D18 ppk1-5FLAG::hphMX6*	This study
YGF592	h^- *ura4-D18 htb1-K119R::kanMX6 ppk1-5FLAG::hphMX6*	This study
YGF594	h^- *ade6? leu1-32 ura4-D18 htb1-G52D ppk1-5FLAG::hphMX6*	This study
YGF595	h^- *ade6? leu1-32 ura4-D18 htb1-P102L ppk1-5FLAG::hphMX6*	This study
YGF599	h^+ *ade6? leu1-32 ura4-D18 htb1-G52D lys1Δ::[pHBKA11-ppk1-5FLAG-hphMX6]*	This study
YGF602	h^+ *ade6? leu1-32 ura4-D18 htb1-P102L lys1Δ::[pHBKA11-ppk1-5FLAG-hphMX6]*	This study
YGF632	h^+ *ade6-M216 leu1-32 ura4-D18 eng1-5FLAG::hphMX6*	This study
YGF633	h^- *ura4-D18 htb1-K119R::kanMX6 eng1-5FLAG::hphMX6*	This study
YGF634	h^- *ade6? leu1-32 ura4-D18 htb1-G52D eng1-5FLAG::hphMX6*	This study
YGF635	h^- *ade6? leu1-32 ura4-D18 htb1-P102L eng1-5FLAG::hphMX6*	This study
YGF644	h^+ *ade6-M216 leu1-32 ura4-D18 aat1-5FLAG::hphMX6*	This study
YGF645	h^- *ura4-D18 htb1-K119R::kanMX6 aat1-5FLAG::hphMX6*	This study
YGF646	h^- *ade6? leu1-32 ura4-D18 htb1-G52D aat1-5FLAG::hphMX6*	This study
YGF647	h^- *ade6? leu1-32 ura4-D18 htb1-P102L aat1-5FLAG::hphMX6*	This study
YGF651	h^+ *ade6-M210 leu1-32 ura4-D18 eng1Δ::kanMX6*	This study
YGF652	h^+ *ade6-M210 leu1-32 ura4-D18 aat1Δ::kanMX6*	This study
YGF653	h^+ *ade6-M210 leu1-32 ura4-D18 SPBC887.17Δ::kanMX6*	This study
YGF655	h^- *ade6? leu1-32 ura4-D18 htb1-G52D lys1Δ::[pHBKA21-5FLAG-hphMX6]) SPNCRNA.1606::[pKANZA11-GFP]*	This study
YGF656	h^- *ade6? leu1-32 ura4-D18 htb1-P102L lys1Δ::[pHBKA21-5FLAG-hphMX6]) SPNCRNA.1606::[pKANZA11-GFP]*	This study
YGF657	h^- *ade6? leu1-32 ura4-D18 htb1-G52D lys1Δ::[pHBKA11-ppk1-hphMX6]) SPNCRNA.1606::[pKANZA11-aat1-GFP]*	This study
YGF658	h^- *ade6? leu1-32 ura4-D18 htb1-P102L lys1Δ::[pHBKA11-ppk1-hphMX6]) SPNCRNA.1606::[pKANZA11-aat1-GFP]*	This study

Table S2. Plasmids used in this study.

Plasmid	Alias	Description	Source
pGF2	pFA6a-htb1-kanMX6	pFA6a containing *htb1* and *kanMX6*	This study
pGF68	pFA6a-htb1-K119R-kanMX6	pFA6a containing *htb1-K119R* mutation and *kanMX6*	This study
pGF73	pFA6a-htb1-G52D-kanMX6	pFA6a containing *htb1-G52D* mutation and *kanMX6*	This study
pGF74	pFA6a-htb1-P102L-kanMX6	pFA6a containing *htb1-P102L* mutation and *kanMX6*	This study
pGF93	pFA6a-htb1-P102S-kanMX6	pFA6a containing *htb1-P102S* mutation and *kanMX6*	This study
pGF98	pFA6a-htb1-D67N-kanMX6	pFA6a containing *htb1-D67N* mutation and *kanMX6*	This study
pGF117	pHBKA11-5FLAG	pUC119-Padh11-5FLAG-hygR-lys1D	Q. Jin
pGF156	pHBKA21-5FLAG	pUC119-Padh21-5FLAG-hygR-lys1D	Q. Jin
pGF186	pFA6a-htb1-G52A-kanMX6	pFA6a containing *htb1-G52A* mutation and *kanMX6*	This study
pGF187	pFA6a-htb1-G52C-kanMX6	pFA6a containing *htb1-G52C* mutation and *kanMX6*	This study
pGF188	pFA6a-htb1-G52S-kanMX6	pFA6a containing *htb1-G52S* mutation and *kanMX6*	This study
pGF189	pFA6a-htb1-G52R-kanMX6	pFA6a containing *htb1-G52R* mutation and *kanMX6*	This study
pGF194	pFA6a-htb1-D67A-kanMX6	pFA6a containing *htb1-D67A* mutation and *kanMX6*	This study
pGF195	pFA6a-htb1-D67G-kanMX6	pFA6a containing *htb1-D67G* mutation and *kanMX6*	This study
pGF196	pFA6a-htb1-D67H-kanMX6	pFA6a containing *htb1-D67H* mutation and *kanMX6*	This study
pGF197	pFA6a-htb1-P102A-kanMX6	pFA6a containing *htb1-P102A* mutation and *kanMX6*	This study
pGF198	pFA6a-htb1-P102T-kanMX6	pFA6a containing *htb1-P102T* mutation and *kanMX6*	This study
pGF210	pFA6a-htb1-G52E-kanMX6	pFA6a containing *htb1-G52E* mutation and *kanMX6*	This study
pGF211	pFA6a-htb1-G52P-kanMX6	pFA6a containing *htb1-G52P* mutation and *kanMX6*	This study
pGF212	pFA6a-htb1-P102I-kanMX6	pFA6a containing *htb1-P102I* mutation and *kanMX6*	This study

pGF213	pFA6a-htb1-D67Q-kanMX6	pFA6a containing *htb1-D67Q* mutation and *kanMX6*	This study
pGF217	pHBKA21-SPBC887.17-5FLAG	pUC119-Padh21-SPBC887.17-5FLAG-hygR-lys1D	This study
pGF218	pHBKA21-aat1-5FLAG	pUC119-Padh21-aat1-5FLAG-hygR-lys1D	This study
pGF219	pHBKA21-eng1-5FLAG	pUC119-Padh21-eng1-5FLAG-hygR-lys1D	This study
pGF220	pHBKA21-ppk1-5FLAG	pUC119-Padh21-ppk1-5FLAG-hygR-lys1D	This study
pGF224	pKANZA11-GFP	pUC119-Padh11-GFP-kanMX6-SPNCRNA.1606	Q. Jin
pGF230	pHBKA11-ppk1-5FLAG	pUC119-Padh11-ppk1-5FLAG-hygR-lys1D	This study
pGF234	pKANZA11-aat1-GFP	pUC119-Padh11-aat1-GFP-kanMX6-SPNCRNA.1606	This study

Table S3. Antibodies used in this study.

Antibody	Description	Source
Actin (rabbit mAb)	IB: 1:10000	Abclonal (AC026)
H2BK119ub (rabbit mAb)	IB: 1:1000; ChIP: 1:200	CST (5546)
H2B (rabbit pAb)	IB: 1:1000	GeneTex (64122)
FLAG (mouse mAb)	IB: 1:1000	Sigma (F1804)
RNA pol II Rpb1 (mouse mAb)	ChIP: 1:500	Sigma (05-623)
IgG-Alexa Fluor Plus 800 (anti-mouse)	IB: 1:10000	Thermo (A32730)
IgG-Alexa Fluor Plus 800 (anti-rabbit)	IB: 1:10000	Thermo (A32735)

Table S4. Primers used in the qPCR assays.

Oligonucleotide	Sequence (5' to 3')	Use
GF85	ATGGTAGATGGAGAAACGGG	Forward primer for ChIP-qPCR *ars2004*
GF86	CACGGCATCTTTCTTCACGA	Reverse primer for ChIP-qPCR *ars2004*
GF352	CCATTGAGCACGGTATTGTC	Forward primer for RT-qPCR and ChIP-qPCR *act1*$^{+}$
GF353	GAGCCTCAGTCAACAAGCAA	Reverse primer for RT-qPCR and ChIP-qPCR *act1*$^{+}$
GF858	GCTCCACAAGTCTCAGAGGCC	Forward primer for RT-qPCR and ChIP-qPCR *aat1*$^{+}$
GF859	CGTCAGCTTCTCTGGCTGG	Reverse primer for RT-qPCR and ChIP-qPCR *aat1*$^{+}$
GF860	GTTTGGCACAATGCTCGCC	Forward primer for RT-qPCR *eng1*$^{+}$
GF861	CGGTGCCGAAATAACTGGCTG	Reverse primer for RT-qPCR *eng1*$^{+}$
GF866	AGTACTGATCGTGCACTACCGG	Forward primer for RT-qPCR and ChIP-qPCR *ppk1*$^{+}$
GF867	GTTAGTGAAGTAGGGGCTTTCGG	Reverse primer for RT-qPCR and ChIP-qPCR *ppk1*$^{+}$
GF868	ACCGCTGCCATTTCTGCTC	Forward primer for RT-qPCR *SPBC887.17*$^{+}$
GF869	TCTCGATAGGAAACACGTCCAGTG	Reverse primer for RT-qPCR *SPBC887.17*$^{+}$
GF978	TTATTGAGTAAGTCAACAATTCAAATTTTCGATTTAGTTAGG	Forward primer for RT-qPCR and ChIP-qPCR *aro1*$^{+}$
GF979	CCTGATTCTACTAGCTGAAGTGCTAAAAAAAATTAC	Reverse primer for RT-qPCR and ChIP-qPCR *aro1*$^{+}$
GF982	GATATCGTCTAGGCCGGCTTAGAG	Forward primer for ChIP-qPCR *eng1*$^{+}$
GF983	AGTCTAAAGGTTCACATCCAGTGTGAAC	Reverse primer for ChIP-qPCR *eng1*$^{+}$
GF986	AGCAAATCTTGAACAATAATAGTATTCTGTCGG	Forward primer for ChIP-qPCR *SPBC887.17*$^{+}$
GF987	ACAGGTTGCGAAATATGTAAGATGAAGC	Reverse primer for ChIP-qPCR *SPBC887.17*$^{+}$

Table S5. The expression of all genes in *htb1-G52D, htb1-G52E, htb1-G52R, htb1-P102L, htb1-P102S, htb1-D67N,* and *htb1-D67H.*

Table S6. The expression of all genes in *htb1-K119R*.

Table S7. The expression of all genes in *htb1-G52D*, *htb1-G52D ubp8*Δ, *htb1-P102L*, *htb1-P102L ubp8*Δ, *htb1-D67N*, and *htb1-D67N ubp8*Δ.

Table S8. DEGs in *htb1-G52D, htb1-G52E, htb1-G52R, htb1-P102L, htb1-P102S, htb1-D67N,* and *htb1-D67H.*

Table S9. DEGs in *htb1-K119R.*

Table S10. DEGs in *htb1-G52D*, *htb1-G52D ubp8*Δ, *htb1-P102L*, *htb1-P102L ubp8*Δ, *htb1-D67N*, and *htb1-D67N ubp8*Δ.
